# Versatility of Induced Pluripotent Stem Cells (iPSCs) for Improving the Knowledge on Musculoskeletal Diseases

**DOI:** 10.3390/ijms21176124

**Published:** 2020-08-25

**Authors:** Clara Sanjurjo-Rodríguez, Rocío Castro-Viñuelas, María Piñeiro-Ramil, Silvia Rodríguez-Fernández, Isaac Fuentes-Boquete, Francisco J. Blanco, Silvia Díaz-Prado

**Affiliations:** 1 Cell Therapy and Regenerative Medicine Group, Department of Physiotherapy, Medicine and Biomedical Sciences, Faculty of Health Sciences, University of A Coruña (UDC), 15006 A Coruña, Galicia, Spain; rocio.castro@udc.es (R.C.-V.); maria.pramil@udc.es (M.P.-R.); s.rodriguezf@udc.es (S.R.-F.); i.fuentes@udc.es (I.F.-B.); 2 Institute of Biomedical Research of A Coruña (INIBIC), University Hospital Complex A Coruña (CHUAC), Galician Health Service (SERGAS), 15006 A Coruña, Galicia, Spain; fblagar@sergas.es; 3 Centro de Investigación Biomédica en Red (CIBER) de Bioingeniería, Biomateriales y Nanomedicina (CIBER-BBN), 28029 Madrid, Spain; 4 Centro de Investigaciones Científicas Avanzadas (CICA), Agrupación estratégica CICA-INIBIC, University of A Coruña, 15008 A Coruña, Galicia, Spain; 5 Tissular Bioengineering and Cell Therapy Unit (GBTTC-CHUAC), Rheumatology Group, 15006 A Coruña, Galicia, Spain

**Keywords:** iPSCs, pluripotency, regenerative medicine, EVs, bone, cartilage, muscle, intervertebral disc

## Abstract

Induced pluripotent stem cells (iPSCs) represent an unlimited source of pluripotent cells capable of differentiating into any cell type of the body. Several studies have demonstrated the valuable use of iPSCs as a tool for studying the molecular and cellular mechanisms underlying disorders affecting bone, cartilage and muscle, as well as their potential for tissue repair. Musculoskeletal diseases are one of the major causes of disability worldwide and impose an important socio-economic burden. To date there is neither cure nor proven approach for effectively treating most of these conditions and therefore new strategies involving the use of cells have been increasingly investigated in the recent years. Nevertheless, some limitations related to the safety and differentiation protocols among others remain, which humpers the translational application of these strategies. Nonetheless, the potential is indisputable and iPSCs are likely to be a source of different types of cells useful in the musculoskeletal field, for either disease modeling or regenerative medicine. In this review, we aim to illustrate the great potential of iPSCs by summarizing and discussing the in vitro tissue regeneration preclinical studies that have been carried out in the musculoskeletal field by using iPSCs.

## 1. Introduction

Musculoskeletal conditions such as osteoporosis, osteoarthritis, fractures, muscular dystrophy and skeletal malformations are the second-greatest cause of disability worldwide [1]. According to the World Health Organization musculoskeletal disorders cost billions of dollars for healthcare annually and are expected to increase further, largely driven by population growth and aging [2]. These conditions affecting tissues within the joints are commonly associated with persistent pain, impaired mobility and function and reduced quality of life and mental well-being, as well as other comorbidities [2,3]. In high-income countries, musculoskeletal conditions are one of the major causes of work loss and early retirement, lost retirement wealth [4] and reduced national productivity [4]. For instance, despite the fact that most of these disorders are not immediately life-threatening, some of them have been proven to have higher mortality rates [2,5]. Although novel pharmacotherapies that improve survival and functioning have been developed for certain muscle diseases, such as Duchenne muscular dystrophy (DMD), spinal muscular atrophy (SMA) or Pompe disease [6], there is no pharmacological treatment that can effectively cure most of these diseases. To date, most of the existing treatments aim to decrease pain and alleviating symptoms [5,7] but the therapeutic outcomes still need to be improved [3].

In order to develop effective treatments, it is of uttermost importance to elucidate the cellular and molecular bases that underlie human diseases through the understanding of critical biological processes [8]. A better understanding of the alterations leading to the development of musculoskeletal diseases is of uttermost importance for the discovery of new therapeutic targets and, therefore, for the development of efficient treatments. A whole range of in vitro and in vivo systems are currently used to study different physiological aspects of both healthy and impaired musculoskeletal tissues [9]. Animal models have hugely contributed to better understand disease mechanisms. However, it is increasingly clear that animal models have limitations in predicting the pathophysiology of many human diseases since they differ from humans in terms of physiology, immune system, inflammation and individual genetic backgrounds [10]. Focusing specifically on degenerative musculoskeletal diseases, disease progression is slower in humans than in animals and pathological changes in animal models may not be entirely consistent with those of the human disease [11]. On the other hand, some compounds have proven to have species-specific toxicity in animals [12] or turned out to be ineffective in human patients after showing therapeutic effects in rodent disease models [13,14]. All those facts demonstrate the need to establish disease models using human samples.

Human primary cell cultures and cell lines have substantially improved our understanding of the mechanisms responsible for many rare and common diseases and have driven the development of novel therapeutic strategies [9]. Although useful, these cells are associated with several drawbacks that hinder the understanding of the molecular factors involved in the early, advanced and final stages of different diseases [9,10]. Human primary cells undergo senescence and have a limited lifespan after isolation and in vitro culture [15]. For instance, human mesenchymal stromal cells (MSCs), often employed as a cell source for orthopedic research, have been described to achieve a maximum of 30–40 population doublings in vitro before they lose their proliferation potential [16]. Other cells such as chondrocytes rapidly lose their molecular signature and quickly dedifferentiate when removed from the joint environment [17,18]. Moreover, primary muscle cells are very sensitive to their physical environment; therefore, these cells are prone to detaching and limiting their mature phenotype on stiff substrates [19]. Additionally, relevant human tissue or cell samples are often difficult to obtain, sometimes requiring invasive surgery or only becoming available post-mortem [15]. Since isolated primary cells cannot be long-term maintained or expanded under conventional culture conditions, immortalized clonal cell lines are a frequently used cell source [16,20,21]. In these lines, cells can be produced in large amounts and grown indefinitely, offering a good tool to explore molecular and biochemical processes [9]. Numerous immortalized cell lines have been generated from MSCs, bone cells, muscle cells and chondrocytes [22,23,24], which have provided valuable information about the processes involved in skeletal development. However, these cell lines may contain genetic and metabolic abnormalities due to their derivation using mainly integrative methods. Thus, these cells would not represent a realistic or ideal drug model for human patients, as they lack the ability to properly recapitulate specific properties of the primary tissue of interest [25]. Alternative strategies to further study and treat musculoskeletal disorders have increasingly involved the use of stem cells in order to replace/repair damaged tissues and improve homeostasis [26,27]. However, the search for cell sources able to restore integrity of musculoskeletal tissues has proven to be challenging [26,27,28]. On one hand, human MSCs are scarce and heterogeneous, have limited differentiation capacity and, as previously mentioned, their proliferation is limited by age [16,29,30,31]. On the other hand, the use of human embryonic stem cells (ESCs) is associated with ethical issues and the risk of rejection when transplanted is an additional disadvantage of using such cells [26,27]. In this context, induced pluripotent stem cells (iPSCs) emerged as a cell source that could potentially overcome the majority of these limitations.

**Figure 1:**
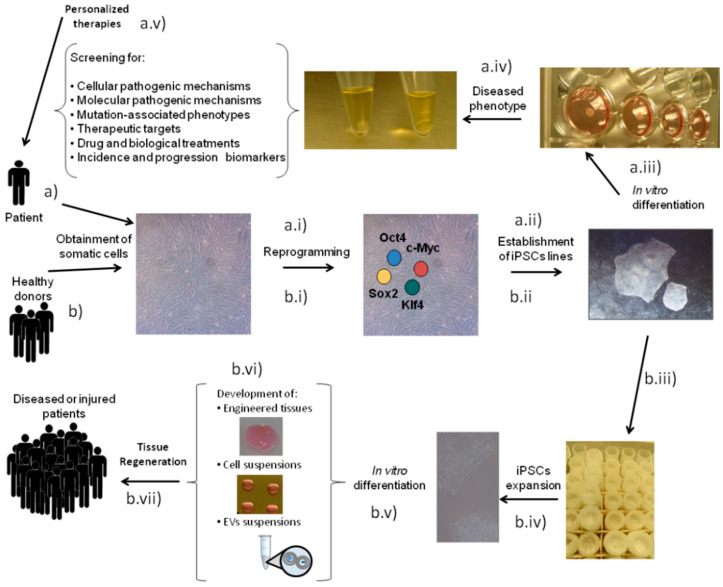
Schematic representation of induced pluripotent stem cells (iPSCs) as a tool for (**a**) disease modeling and (**b**) regenerative medicine. For disease modeling purposes, somatic cells are obtained from a patient and reprogrammed (a.i) to establish the iPSC line (a.ii). In vitro differentiation (a.iii) is necessary to obtain the diseased phenotype (a.iv) that allows for the set-up of disease models and drug discovery platforms (a.v). For regenerative medicine approaches, somatic cells can be obtained from healthy donors and reprogrammed (b.i) to establish iPSC lines (b.ii), which are deposited in a bank (b.iii). iPSCs from banks only need to be expanded (b.iv) and differentiated in vitro (b.v) to develop the biological treatment (b.vi) that can later be used for tissue regeneration (b.vii) in a group of diseased or injured patients.

iPSCs were generated over a decade ago by Takahashi and Yamanaka, who succeeded in reprogramming mouse tail-tip fibroblasts by means of retroviral introduction of only four transcription factors: octamer-binding protein 3/4 (Oct3/4), SRY (sex-determining region Y)-box 2 (Sox2), Krüppel-like factor (Klf4) and c-Myc [32,33]. iPSCs display almost analogous characteristics to ESCs, providing researchers with an unlimited cell source capable of differentiating into any cell type of the body [3,33]. These cells can be employed as tools to dissect developmental mechanisms but also to trigger a general interest for the advancement of new human disease models and enhanced platforms for drug discovery [34,35,36] (Figure 1a) due to their ability to supply unlimited quantities of clinically relevant cell types and their potential to be derived from easily accessible cells [36]. iPSCs also hold great potential for regenerating damaged tissues/organs (Figure 1b) and restoring functions impaired by diseases [3]. In this regard, the notion of personalized medicine using iPSCs is very attractive and, as such, has been explored to some extent, especially during the first years after their generation. However, nowadays that notion is starting to be rejected, as the generation of patient-specific iPSCs is time-consuming and very costly [37]. Therefore, future therapies based on this technology could involve the use of allogenic iPSCs and/or iPSC-derived cells or even iPSC secretome [38,39,40,41,42] (Figure 1b).

Although the potential of iPSCs for tissue regeneration, disease modeling and drug screening has been largely recognized, the findings of iPSC research to date are mostly focused on the neurology, cardiology and hematology fields [3]. Nonetheless, in recent years there has been a growing interest in using cellular reprogramming as a tool to study pathogenesis of mutation- and ageing-associated musculoskeletal disorders and to explore their potential for tissue repair. Recent work by Li et al. [3] reviewed the orthopedic application of iPSCs in ageing-associated musculoskeletal disorders, offering a useful recapitulation of the existing protocols to differentiate iPSCs into musculoskeletal cell types. In this review, we complement the aforementioned work by providing a thorough description of the already established iPSC-based congenital and ageing-associated musculoskeletal disease models, paying special attention not only to cartilage, muscle and bone but also to the nucleus pulposus. In addition, the application of iPSC-derived cells to tissue engineering approaches is discussed. Efficient and robust differentiation of iPSCs into tissue-specific cell types is crucial both for disease modeling and for regenerative medicine. Should the reader seek a more detailed explanation about iPSC differentiation protocols, please see the previously published studies on osteogenic [3,30,43,44,45], chondrogenic [3,27,30,46], nucleus pulposus [47,48,49] and/or myogenic [3,30,50] differentiation of iPSCs.

## 2. Disease Modeling

As previously mentioned, the use of primary and immortalized cell lines as in vitro models for musculoskeletal diseases presents intrinsic drawbacks. Although not exempt from limitations, the use of iPSCs has proven to be the best alternative to overcome the problems associated with these conventional cell-based in vitro models [51]. Reprogramming technology has provided researchers with easy-access human pluripotent stem cells, which are capable of self-renewal and have the potential to differentiate into any cell type through the use of differentiation protocols to generate specific target cells [15]. In theory, almost every human disease can be modeled by using iPSC platforms, including monogenic, chromosomal and complex disorders, epigenetic disorders and disorders that appear early or late in life [51]. Currently, national and international initiatives are establishing repositories of human iPSCs as models for human disorders [52].

The first models of disease employing iPSCs were reported in 2008, when Park and colleagues produced the first large repository of disease-specific iPSCs [53]. Later on, in 2009, studies on iPSC-based models of SMA developed by Ebert et al. [21] demonstrated that the phenotype of diseased cells could be recapitulated in a Petri dish. Since then, a wide variety of studies have reported the derivation of specific cell types from iPSCs, generating robust and reproducible phenotypes in vitro that reflect diseases intrinsic to the cellular level [35]. Specifically, evidence that reprogrammed cells can be differentiated into osteoblasts [54,55,56], chondrocytes [57,58,59,60], myoblasts [61], nucleus pulposus cells [47,48,49] and tenocytes [62] has recently impacted musculoskeletal research and changed orthopedic medicine. Nevertheless, the use of iPSCs for studying the wide variety of musculoskeletal conditions has been explored to a limited extent (Table 1) in comparison with conditions affecting nervous or cardiac pathologies, even when investigating genetic disorders where symptoms affect several organs or systems [63,64,65,66].

On the other hand, the more widespread availability of gene editing techniques makes it easier to generate in vitro iPSC-based models, which allow not only to better study the pathology of musculoskeletal disorders and their associated phenotypes but also to correct pathogenic mutations in patient-derived iPSCs. Common technologies for genome-editing are meganucleases [67], zinc finger nucleases (ZFNs), transcription activator-like effector nucleases (TALENS) and clustered regularly interspaced short palindromic repeats/CRISPR-associated protein 9 (CRISPR/Cas9) [67,68]. In particular, CRISPR/Cas9 is widely used because of its specificity, although the possibility of producing off-target effects should not be ruled out. Additional advantages of this system include its easier design and quicker generation when compared with other systems [67,69]. In the sections below, we include several examples that illustrate the substantial contribution of gene editing techniques to the understanding of musculoskeletal disease pathology and the role of specific mutations in disease development. Furthermore, it is worth highlighting that integrated gene-edited lines along with isogenic controls can constitute useful testbeds for preclinical analysis of therapeutic efficacy and toxicity of dedicated drugs and compounds [70].

### 2.1. Modeling Bone Diseases with iPSCs

Bone disorders have been defined as a group of varied acute and chronic traumatic, degenerative, malignant or congenital conditions affecting the musculoskeletal system [43]. Research on new treatments for bone disorders has greatly expanded in recent years thanks to the introduction of reprogramming methods and the subsequent production of iPSCs, which provides the possibility to create human-specific models.

Bone is a dynamic tissue that is continually remodeling through coordinated osteoclast-mediated destruction followed by osteoblast-mediated reconstruction [24,76]. This balance between bone reabsorption and bone formation is crucial to preserve the mechanical integrity of the skeleton. In osteoporosis, for example, the balance is impaired and more bone is reabsorbed than formed, which results in the loss of bone mass and a decrease in bone density [43,110]. The damage or loss of bone tissue and dysosteogenesis still represents a serious problem in orthopedics [110]. A thorough understanding of the factors, mechanisms and interactions that regulate the differentiation of each of the cell types found in bone tissue is central to the design of therapeutic strategies to treat bone diseases [24]. Differentiation of iPSCs into osteoblasts [54,55] is expected to be useful for this purpose. Many genetic bone disorders have limited treatment possibilities due to the absence of appropriate animal models and the inaccessibility of native bones but iPSC-derived disease models from specific patients can allow us to understand the origins and pathologies of these diseases [43]. In this sense, human iPSCs have been employed as disease models to better understand several diseases that affect bone or bone pathologies such as Marfan’s syndrome (MFS), Andersen’s syndrome (AS), fibrodysplasia ossificans progressiva (FOP), osteogenesis imperfecta (OI) or other rare disorders that affect the musculoskeletal system.

MFS is an autosomal dominant genetic disorder of the connective tissue caused by mutations in the FIBRILLIN-1 (FBN1) gene and associated with skeletal deformities (disproportionate growth, scoliosis), among other symptoms [63,64,65]. Klein et al. [63] reprogrammed skin fibroblasts derived from an MFS patient and demonstrated the presence of a mutation in the FBN1 gene in the established iPSC line by sequencing. Park et al. [64] went a step further and derived iPSCs from an MFS patient with an FBN1 mutation and corrected it, thereby generating isogenic “gain-of-function” control cells for the parental MFS-iPSCs. With their experiments, they showed that phenotypic changes associated with MFS could be recapitulated in the osteogenic-like cells derived from MFS-iPSCs: reduced osteogenic differentiation and microfibril formation in MFS-iPSCs, which are features associated with MFS and with FBN1 function [64]. MFS-iPSCs also showed lower sensitivity to carbachol compared with isogenic control cells as demonstrated by reduced contractility and reduced response in a Ca^2+^ influx assay. Furthermore, using TALEN-mediated correction of the mutation in MFS-iPSCs, they demonstrated that it was possible to rescue the compromised osteogenenic differentiation [64], thus proving the causal role of that specific mutation in the pathogenesis of MFS. Similarly, Pini et al. [71] reprogrammed skin cells carrying an AS-associated mutation. AS is a rare disorder characterized by bone developmental defects, among other symptoms. They showed that osteogenic machinery was hastened in AS-iPSCs, according to the expression of two master genes for osteoblastic differentiation (RUNX2 and OSTERIX), strongly suggesting that the generated cells could be a good model to better understand AS pathophysiology [71].

Panicker et al. [66] using iPSCs shed light on the mechanisms that could lead to GD. This disease is developed due to alterations in the gene that encodes acid β-glucocerebrosidase and causes bone abnormalities in patients. By generating iPSCs from patients with GD and differentiating them into osteoblasts, they found that these cells had developmental defects and lysosomal abnormalities that interfered with bone matrix deposition, thus discovering a new therapeutic target for the treatment of bone abnormalities in GD [66].

FOP, characterized by progressive ossification in soft tissues, is another rare genetic disorder that has been ‘modeled in a dish’ employing patient-derived iPSCs [111]. Matsumoto et al. [72] created iPSCs derived from normal and FOP dermal fibroblasts and tested their ability to contribute to endochondral bone formation. Interestingly, they observed that FOP-iPSCs showed increased mineralization and enhanced chondrogenesis in vitro when compared with control iPSCs. Additionally, they used these cells as a platform for drug screening and reported that abnormal bone growth could be suppressed with DMH1, a small-molecule inhibitor of BMP signaling [72], a finding that could help guide the future development of drugs to treat for this condition.

The generation of iPSCs from patients with osteopetrosis [74,75], an autosomal condition caused by defects in osteoclast formation and function, has also opened up new ways to identify osteoclast defects leading to osteopetrosis [40,74,75].

Osteoporosis still represents a significant public health problem affecting a broad spectrum of the elderly population. To our knowledge, there are no studies modeling osteoporosis per se but rather modeling rare diseases where osteoporosis is part of their clinical manifestation. That is the case of the iPSCs generated from patients with Turner syndrome (TS) [79], a rare disease caused by a monosomy X. TS and healthy iPSCs were differentiated into osteoblasts and osteoclasts, with abnormal gene expression in TS iPSC-osteoclasts. The knowledge gained from studies on OI can also provide clues about the genetics of osteoporosis [24]. Recently, it has been published that it is also possible to model the various cell phenotypes (characterized by altered expression of COL1A1 and ALPL and decreased levels of calcium deposition) observed in bone diseases such as OI, a syndromic disease characterized by fragile bones in which clinical phenotypes range from perinatal lethality to osteoporosis [76]. Moreover, an OI iPSC line has been developed from a patient with a lethal perinatal form of OI caused by a heterozygous single mutation in the COL1A1 gene. An isogenic control line was also generated using CRISPR/Cas9 gene editing [77]. Deyle et al. [78] went a step further and obtained MSCs from OI patients with either COL1A1 or COL1A2 mutations. These mutations were inactivated by adeno-associated virus (AVV)-mediated gene targeting before iPSC reprogramming and the transgenes were removed with Cre-recombinase after iPSC-MSC derivation. These MSCs were able to differentiate into osteoblasts both in vitro and in vivo [78]. Both studies developed lines expected to be useful for exploring OI mechanisms and therapeutic approaches.

Li-Fraumeni syndrome (LFS) is another rare genetic condition associated with bone malignancy, osteosarcoma being one of the main cancer types seen in LFS patients. Lee et al. [80] generated an LFS disease model established from patient-derived iPSCs and found that LFS iPSC-derived osteoblasts recapitulated clinical osteosarcoma features. These features included defective osteoblastic differentiation (lower expression of osteogenic markers compared with healthy controls) and gain of tumorigenic ability (impaired transcriptional activity of p53), demonstrating that iPSCs can serve as an invaluable in vitro disease model to elucidate osteosarcoma etiology [80]. Zhou et al. [81] also recapitulated osteoblastic tumorigenesis after differentiation from LFS iPSC-MSCs. They injected LFS iPSC MSC-derived osteoblasts subcutaneously in NU/NU mice and observed immature osteogenic tumor formation.

Interestingly, in vitro iPSC-based models of bone-affecting diseases are scarce and are usually reported by only one single research group, which makes it difficult to compare protocols and phenotypes. AS [112] and GD [113,114] models have been generated by other groups, although with the purpose of studying cardiac or neural diseases. Nonetheless, all of these findings derived from iPSC-based disease models and subsequent in vitro experimentation provide a proof-of-concept that the use of human iPSCs for bone research is definitely improving our understanding about human skeletal disorders and how to correct them at the molecular level and has allowed for the discovery of new therapeutic targets and even of potential treatments.

### 2.2. Modeling Cartilage Diseases with iPSCs

Apart from bone diseases, patient-specific iPSCs have been created in order to model cartilage diseases. Refined protocols for chondrogenically differentiating iPSCs have paved the way to developing in vitro models for cartilage diseases, as well as new screening platforms for testing new drugs based on their use. Among previously published studies, several groups have focused on modeling cartilage diseases produced by monogenic mutations but studies modeling complex cartilage diseases affected by several factors are also starting to arise.

Xu et al. [82] employed the iPSC technology to elucidate the cellular phenotype of familial osteochondritis dissecans, a joint disease characterized by the separation of articular cartilage from subchondral bone and that usually leads to the development of osteoarthritis (OA). The analysis of the phenotype of differentiated cells revealed an abnormal intracellular processing and extracellular distribution of aggrecan, a major proteoglycan of the articular cartilage produced by chondrocytes. As a consequence, synthesis and assembly of the entire cartilage extracellular matrix (ECM) was impaired. Moreover, they also found that composition of the ECM in patients’ iPSC-derived chondrocytes (poor in aggrecan and rich in asporin, mimecan, fibronectin, matrilin-3, COMP, tenascin-C and perlecan) reflected the changes observed in advanced OA, presenting further evidence of the association between familial osteochondritis dissecans and early-onset OA [82].

The understanding of the pathophysiology of neonatal-onset multisystem inflammatory disease (NOMID) arthropathy has also improved thanks to iPSC technology. NOMID arthropathy is an auto-inflammatory disease caused by NACHT, LRR and PYD domains-containing protein 3 (NALP3) gene mutations. Yokoyama et al. generated iPSCs derived from NOMID patients and produced chondrocyte-like tissues with mutant and wild-type NLRP3. Interestingly, they revealed a previously unidentified connection between the inflammasome-associated molecule NLRP3 and the master regulator of chondrocyte differentiation SOX9. Furthermore, they showed that SOX9 was overexpressed via the cAMP/PKA/CREB signaling pathway in chondrocytes with disease-causing mutations in NLRP3 [59]. Surprisingly, Kawasaki et al. [83] also established iPSCs from a NOMID patient but lacking the NLPR3 mutation and found a heterozygous NLRC4 mutation for which CRISPR/Cas9 knockout reversed the pathogenic phenotype (anomalous cytokine profile).

FGFR3 chondrodysplasia is another monogenic cartilage disorder that was modeled using iPSC lines derived from patients [84,85], which showed recapitulation of the pathology in vivo (small hypertrophic chondrocytes) and in vitro (low presence of glycosaminoglycans in the ECM, decreased proliferation and increased apoptosis) and demonstrated the effectiveness of an FGFR inhibitor as a potential treatment for the disease [84,85].

In addition to being used for genetic diseases, patient-specific iPSCs have been also generated from patients with complex diseases such as rheumatoid arthritis (RA) [86] and OA [86,88,89]. In these studies, iPSCs were generated from patients with OA or RA in order to check whether human synoviocyte- or chondrocyte-derived iPSCs showed differences in their chondrogenic capacity. These investigations coincide in demonstrating that iPSC-based models recapitulated key changes in chondrocyte phenotype and matrix production found in OA (hypertrophy and loss of proteoglycans and collagens in the ECM) or RA cartilage (high inflammatory environment and overexpression of proteases), providing an alternative way to develop research on both of these diseases. Finally, it is worth pointing out that access to proper healthy controls for studying OA and/or RA is limited due to ethical considerations [9]. A recent advance in this regard has consisted of the generation of an iPSC line derived from a patient with no rheumatic diseases, as proved by radiographic information [87]. This control line may help researchers to compare phenotypes and, ultimately, to reach firmer conclusions when modeling rheumatic diseases.

Overall, these recent advances set the basis for the generation of a new tool for the study of cartilage disorders. The possibility to model ‘disease in a dish’ by means of iPSC technology opens the door to the development of novel therapeutic compounds and, more importantly, to an improved understanding of cartilage diseases.

### 2.3. iPSC-Based Disease Modeling for Skeletal Muscle

In addition to bone and cartilage diseases, patient-derived iPSCs, which possess the ability to differentiate into myogenic progenitor cells followed by myotubes, can be a useful tool for drug screening and modeling of skeletal muscle diseases [115]. For these purposes, an efficient and reproducible myogenic differentiation method is required. The possibility to generate patient-specific iPSCs, which can be subsequently differentiated into myogenic cells, have helped researchers to establish disease models of skeletal muscle disorders, such as muscular dystrophies (MDs) or Miyoshi myopathy (MM), among others.

So far, different groups have demonstrated the suitability of iPSCs to derive large quantities of myogenic precursors [116] and to model MDs as reviewed elsewhere [5]. MDs are a spectrum of muscle disorders caused by a number of gene mutations [117]. Abujarour et al. [95] derived iPSCs from patients with either Duchenne or Becker MD and found that MD-iPSCs showed aberrant expression of inflammation genes and collagens, BMP/TGFβ signaling and reduced myotube formation compared with control iPSCs, therefore reflecting the disease-specific background of the cell lines. Moreover, they showed that dystrophic iPSC-derived myoblasts had the potential to functionally respond to hypertrophy-inducing factors Wnt7a and Insulin Growth Factor 1 (IGF-1), which are under investigation as potential treatments for MDs in preclinical and clinical studies, respectively [95]. As mentioned above, genome editing technologies such as CRISPR/Cas9 allow for the correction of genetic mutations, raising hope for in vivo genome therapy, which offers a fundamental cure for these daunting inherited MDs. Current applications of iPSC as MDs disease models for studies on pathogenic mechanisms and therapy development have been reviewed elsewhere [117]. Choi et al. [96] also generated a human DMD model using iPSCs and showed that DMD-iPSC-derived myoblasts exhibited disease-related phenotypes, including altered transcriptional profiles, aberrant intracellular signaling and defective myotube formation. They additionally described how these DMD phenotypes could be partially reversed by genetic and pharmacological approaches [96]. Ferrari et al. [97] also generated two DMD iPSC lines from two patients with different mutations in the DYSTROPHIN gene, relevant to study its role in skeletal muscle and other tissues, as well as to asses therapies based on gene editing for this disease. Kyrychenko et al. [98] described different strategies for CRISPR/Cas9 genome editing to correct mutations in the ABD-1 region of the DMD gene. Hypothetically, the use of autologous iPSC-derived myogenic progenitor cells in which the DYSTROPHIN gene is corrected by CRISPR/Cas9 technology could regenerate muscles in patients with DMD [118].

Myotonic dystrophy 1 (DM1) is a multisystem disorder primarily affecting the central nervous system, heart and skeletal muscle. Recently, iPSC-based DM1 models were established using the PAX7 conditional expression system. These cells were differentiated into myogenic progenitors and, subsequently, terminally differentiated into myotubes. Interestingly, DM1 iPSCs differentiated into the myogenic lineage recapitulated the molecular events associated with the DM1 phenotype, such as the splicing disruption of MBNL1 target genes [99]. Dastidar et al. [100] reported CRISPR/Cas9-mediated excising of a CTG-repeat expansion in the myotonic dystrophy protein kinase (DMPK) gene in DM1 patient-derived iPSCs and iPSC-myogenic cells. Normalization of splicing pattern or intracellular localization of some proteins was reported but the correction of the diseased phenotype of iPSC-myogenic cells was not tested [100].

The term “laminopathies” comprehend 16 rare disorders that have mutations in LMNA as a common characteristic. Four out of 16 disorders affect skeletal muscle as well as other tissues: dilated cardiomyopathy (DCM), Emery-Dreifuss muscular dystrophy (EDMD), limb-girdle muscular dystrophy (LGMD) type 1B (LGMD1B) and LMNA-related congenital muscular dystrophy (L-CMD) [94]. The exact pathophysiology of these laminopathies remains unclear, compounded by the rarity of these disorders. Investigations developed by Steele-Stallard and colleagues [94] on these skeletal muscle laminopathies by generating iPSC-based models of L-CMD and LGMD1B recapitulated in vitro disease-associated phenotypes, including abnormal nuclear shape and mislocalization of nuclear lamina proteins. Maffioletti et al. [119] demonstrated that artificial muscles could be obtained using iPSCs from DMD, LGMD2D and LMNA-related muscular dystrophies. These investigations, combined with the proliferation capacity of iPSCs, provide hope to bypass one of the major obstacles when studying these disorders, which lies in obtaining the appropriate number of ‘diseased’ cells to carry out in vitro studies. In addition, other investigations have focused on other affected tissue instead of skeletal muscle [101,102] and therefore have not been included or thoroughly described in this review.

MM is a congenital distal myopathy caused by defective muscle membrane repair as a result of mutations in the DYSFERLIN gene. iPSC-based MM disease models developed by Tanaka and colleagues [90] were able to recapitulate defective membrane repair in derived myotubes. Furthermore, these researchers demonstrated that it was possible to rescue the phenotype of MM by overexpressing DYSFERLIN using plasmid transfection [90]. Avoiding the use of genetic manipulation, Kokubu et al. [91] developed a drug screening platform using iPSC-derived myocytes. They found that nocodazole was able to increase DYSFERLIN expression in cells. The same mutation in the DYSFERLIN gene was found in another muscular dystrophy: limb-girdle muscular dystrophy type 2B (LGMD2B) [120]. Turan et al. [92] employed the CRISPR/Cas9 gene editing system to correct DYSFERLIN and α-sarcoglycan mutations in LGMD2B and LGMD2D patient-derived iPSCs, respectively. They suggested that “corrected” iPSCs could be used as therapeutic agents against these diseases. The same approach was employed by Selvaraj et al. [93] to correct limb-girdle muscular dystrophy type 2A (LGMD2A) patient-derived iPSCs, demonstrating the rescue of the CALPAIN 3 gene in both in vitro and in a mouse model.

In addition to MDs, there are many other diseases affecting the muscular system that have been modeled by means of iPSCs, such as carnitine palmitoyltransferase II deficiency [103], valosin containing protein disease [104,105], Pompe disease [106,107,108,109,121] and other multisystem disorders [122]. Overall, patient-derived iPSCs are useful tools for modeling skeletal muscle diseases. In addition, many efforts have been made to correct the causative mutations of these diseases, especially in MDs. Results obtained so far indicate that the correction of these mutations by gene editing in patient-derived iPSCs is feasible and that autologous, “corrected” iPSCs are a potential treatment for MDs. However, as this type of personalized medicine is not currently achievable (due to costs, safety, etc.), allogenic healthy iPSCs can be an alternative.

### 2.4. iPSC-Based Disease Modeling for Degenerative Disc Disease

To our knowledge, there is only one study that generated iPSCs from patients with degenerative disc disease [49]. In this work, iPSCs were generated from nucleus pulposus cells and were differentiated back into nucleus pulposus cells after reprogramming. Although this study provided the proof-of-concept that iPSCs can be derived from patients with degenerative disc disease, further studies are needed to find out whether it is possible to recapitulate the specific phenotype in vitro and to understand the mechanisms underlying the disease.

## 3. iPSCs in Regenerative Medicine

Bone, intervertebral disc and muscle all have the inherent ability to regenerate after injury, unlike cartilage, tendon and ligaments, which either have almost no intrinsic repair potential or heal with inferior properties. However, while bone and skeletal muscle can easily regenerate, they usually fail to do it when a large volume of diseased tissue is involved [123]. Moreover, the loss of progenitor cells in the intervertebral disc with aging [124,125] or a non-favorable environment for bone-resident MSCs [126] can lead to the failed tissue repair. Different approaches and strategies were largely developed by either cell therapy (using cells) or tissue engineering (using cells, scaffolds and biofactors) [127] to try to solve defective tissue regeneration.

Progenitor cells, responsible for the renewal of most normal tissues in the body, have largely been proposed as the alternative to adult mature cells in therapeutic strategies to treat musculoskeletal disorders [127]. The use of adult cells presents several challenges, such as variation among donors, dependence of functionality on the donor’s age and health condition, heterogenicity, cell scarcity and the use of invasive techniques to obtain them [3,31,128]. Nevertheless, current cell-based therapies have not demonstrated full regeneration of most damaged tissues [30,31], except for cell-based therapy for corneal burns [129] and the gold standard curative bone marrow transplantation [130]. Nevertheless, the success of these cell-based therapies drives the development of more advanced, innovative cell therapies such as iPSC-based therapies.

New therapeutic strategies have increasingly involved the use of stem cells in order to replace/repair damaged tissues and improve homeostasis [26,27]. iPSCs’ capacity for self-renewal without losing their pluripotency allows obtaining high numbers of cells to differentiate into the desired cell type. Protocols for iPSCs differentiation into musculoskeletal tissues follow at least one of these basic strategies [30,44,46,50]: co-culture with primary cells, derivation using combinations of growth factors or small molecules, differentiation through progenitors (MSCs, osteoblasts or myoblasts) and/or differentiation through embryoid body (EB) formation. In the case of myogenic differentiation, EB formation was proved to be less efficient than for chondrogenesis and osteogenesis [30]. Other approaches combined seeding or bioprinting iPSCs on bioactive scaffolds [45,50,131] or magnetic cell delivery [132]. Better differentiation results with good phenotypic stability were reported when subcutaneously injecting iPSCs cells into animals [60,133,134,135]. However, preclinical studies using iPSCs as therapeutic cells either for joint lesions or for joint-affecting diseases are scarcer than in other fields of research.

Below, we provide examples of iPSC-based repair therapies for bone, cartilage and muscle (Table 2).

MSCs derived from iPSCs were found to be comparable to adult MSCs in terms of multipotency even when derived using different protocols [134,138,145]. Osteochondral and bone defects treated with iPSCs or differentiated iPSCs on scaffolds did not show differences when compared with adult MSCs transplanted using the same procedures [44,137,142]. Jungbluth et al. [142] derived MSCs from iPSCs and combined them with calcium phosphate granules to treat critical bone size defects in minipigs. In this case, iPSC-MSCs on scaffolds improved the osseous consolidation compared with scaffold alone but no significant differences were found when compared with a composite with autologous bone marrow and calcium phosphate. As expected, the highest repair was found using autologous bone, which is the gold standard in bone regeneration. Kouroupis et al. [145] tested the MSCs vs. human iPSC-MSCs potential to repair anterior cruciate ligament injuries in swine after osteogenic and ligament induction of both MSCs on Leeds-Keio scaffold and both cell types showed good ligament repair after four months.

Differentiation of iPSCs into chondrocytes showed better repair capacity of osteochondral lesions than iPSCs or iPSC-MSCs in independent studies [136,138]. Uto et al. [137] cultured iPSCs in chondrogenic media and embedded them in atelocollagen before soaking them in beta-tricalcium phosphate (TCP)/poly-L-lactic acid (PLLA) scaffolds and implanted them in osteochondral defects in miniature pigs. After eight weeks, the formed neo-tissue was similar to the one formed by adult MSCs and both were better than TCP scaffolds alone. Nejadnik et al. [138] injected MSCs and chondrocytes derived from iPSC pellets on polyethylene glycol (PEG) and chondroitin sulfate (CS) methacrylate scaffolds to osteochondral defects in rats, obtaining better repair results with iPSC-chondrocytes. In addition, osteoarthritis-induced rats treated with knee injections of iPSCs and chondrocytes derived from iPSCs showed better repair when using iPSC-chondrocytes but osteoarthritic lesions could still be observed with both treatments after 15 weeks [136].

Other works did not compare iPSCs with adult MSCs or autologous grafts for osteochondral repair. Xu et al. [139] implanted human iPSC-MSCs on poly lactic-co-glycolic acid (PLGA) scaffolds in osteochondral defects in rabbits and, after six weeks, histology showed that, although repair was not very successful, those treated with iPSC-MSCs showed better results than those untreated or treated only with scaffolds. They confirmed that subchondral bone formation was poor in all the groups. Ko et al. [140] differentiated iPSCs chondrogenically in micromass or alginate hydrogels and transplanted them into osteochondral defects in rats. After 12 weeks, successful repair of cartilage defect in vivo was detected in those treated with human iPSCs in comparison with those untreated and those treated with hydrogel alone but the tissue formed presented lower levels of glycosaminoglycans (GAGs) than the surrounding native cartilage. Conversely, Yamashita et al. [60] derived human iPSCs into cartilaginous particles through mesendoderm-chondrogenic differentiation before transplanting them in osteochondral defects in rats and minipigs. After four weeks, good quality cartilage-like neotissue was formed in both in vivo models.

In bone regeneration, it is also usual to differentiate iPSCs into osteoblasts prior to transplantation. iPSCs obtained from patients with craneodysplasia were differentiated into osteoblasts before transplantation in bone defects in rats [141]. The authors showed that bone regeneration improved when using osteoblasts with the reverted mutation, while mutated osteoblasts showed poor regeneration. Another study tested the combination of two cell types derived from iPSCs in the same scaffold for bone regeneration [148]. iPSCs were differentiated into osteoblasts and osteoclasts and sequentially seeded on hydroxyapatite (HA)-PLGA/PLLA scaffolds, resulting in a good mature bone-like tissue after subcutaneous transplantation in rodents.

Studies about skeletal muscle regenerative medicine using pluripotent stem cells have been mainly performed using ESCs [50]. Some studies have used iPSCs differentiated through genetic manipulation for implantation in dystrophic mice. Goudenege et al. [143] used iPSCs obtained from DMD patients that were differentiated into myogenic-like cells, forcing the expression of MyoD and then injected into damaged muscles in mice. While their repair capacity was not tested, cells were found to fuse with existing muscle fibers at higher rates than myogenic-like cells derived from ESCs or control myoblasts after four weeks [143]. Another study used myogenic-like progenitor cells obtained from iPSCs cells with inducible Pax7 expression, which were transplanted into dystrophic mice [144]. Engraftment with treated muscles and improved contractility was observed after four weeks but these results were not consistent across all the clones used.

In the case of degenerative disc disease, several studies have focused on the resettlement of cells that had been lost in the intervertebral disc by injecting iPSCs [146] differentiated into nucleus pulposus [48] or notochordal cells [147], with very promising outcomes. These studies used different hydrogels with and without growth factors.

In general, all these studies suggest that MSCs derived from iPSCs show similar repair results than those obtained using adult MSCs. Moreover, further differentiation into mature cells showed better outcomes than intermediate progenitor-derived cells from iPSCs. However, improved differentiation protocols seem to be necessary in order to obtain better regeneration results in preclinical models.

## 4. Limitations, Challenges and Future Directions of iPSCs Applications

Despite the valuable potential of iPSCs, there are some limitations that need to be accounted for when pursuing applications for disease modeling and regenerative medicine. Some of the drawbacks affecting the research on iPSCs are mainly related to cell differentiation, both in terms of differentiation potential varying among cells [149] and of obtaining fully differentiated cell populations that remove the risk of teratoma formation. The differentiation potential of iPSC lines may vary depending on the donor cell source due to incomplete resetting of methylation patterns during reprogramming, which is known as epigenetic memory [36,150,151]. On the positive side, current advancements on the epigenetic memory of iPSCs allow for the exciting opportunity of using reprogramming strategies to reset methylation patterns associated with cellular aging [152,153]. These findings have led into new research ventures to study how reprogramming may rejuvenate ageing cells and to develop iPSC-based therapeutic interventions for age-related diseases [3]. It is also worth considering that heterogeneity among iPSC lines due to inter-individual variability may mislead data interpretation when employing healthy donor-derived iPSCs as controls for disease modeling. To overcome this problem, patient-specific isogenic iPSC lines may be generated by gene editing approaches from preexisting iPSC lines [36,68]. Currently, it is even possible to reprogram cells into iPSCs and generate CRISPR/Cas9-dependent insertions/deletions simultaneously, which allows for the rapid generation of genetic disease cell models with isogenic controls [154]. In addition, these gene editing strategies have proven useful to overcome the problems related to the intrinsic defective nature of patient-derived iPSCs [155] but further investigations towards the refinement of these methodologies are still needed to solve the difficulties and challenges faced by this type of technology in clinical settings, such as its low efficiency and off-target effects [67,156].

The current scarcity of robust and efficient differentiation protocols cannot be underestimated either [157]. Immature functional and structural properties of differentiated iPSCs have been described in other fields [158] and, consequently, obtaining a restored and well-differentiated functional tissue using cell therapy is still one of the biggest issues that need to be addressed within the field of regenerative medicine. In addition, there is a lack of consensus on procedure standardization. Different studies are currently being carried out aiming to establish, for example, the proper number of doses and/or cells per treatment necessary to achieve an optimal functional effect [155] and to improve the so far poor cell engraftment [159]. Once these issues are solved, translational research will be the way forward; however, as discussed above, the autologous use of iPSCs for regenerative medicine purposes is still under debate due to the high costs associated with their generation [37]. On the other hand, the potential immune reactions derived from the use of allogenic cells needs to be accounted for as well [155,160]. In this sense, the generation of iPSCs’ banks with different human leukocyte antigen (HLA) types that can match most of the population [161,162] and the modification of iPSCs to make them hypoimmunogenic [163] are two major steps towards universal transplantation. Nevertheless, low differentiation efficiencies are hampering the translational application of iPSC-based basic research findings, because the presence of undifferentiated cells can contaminate the final, fully differentiated cell population, thus representing a safety issue due to the risk of teratoma formation [42,164]. Even when no teratomas were found in several in vivo studies using different types of animals [60,137,139,140,145,165], there is still some concern [166]. Some studies reported absence of teratomas when iPSCs were transplanted in vivo as differentiated MSCs [139,142,145], lineage-differentiated cells [60,140,143,165] or even iPSCs seeded on scaffolds [137]. However, other studies have shown teratoma formation in some animals after treatment with iPSCs seeded on collagen and implanted in osteochondral defects in rats [167] or after one week of differentiation before implantation in mice [164]. Importantly, both studies were performed using iPSCs reprogrammed using integrative retroviral transduction, which have a higher risk of teratoma formation [128].

Safety concerns and new advances in the study of stromal and stem cell secretome have opened the door to new cell-free tools for regenerative medicine, particularly the therapeutic potential of extracellular vesicles (EVs) [168,169]. Initially, cell therapies were thought to work only as cell engraftment within a damaged tissue, in which cells had to survive long enough after administration to achieve maximum effect [155,170]. Nowadays, it is increasingly accepted that the secretome of exogenous cells induces most of the healing process in endogenous cells [41,42,170,171]. Low cell engraftment and survival of implanted cells highlighted this notion [123].

EVs, including exosomes and microvesicles, are cell-derived membrane-enclosed signaling organelles that mediate intercellular communication [172]. EVs are produced and secreted by almost all cell types and contain proteins, mRNAs, microRNAs and lipids in their cargo. [173,174]. The most commonly studied EVs in therapy are exosomes, which include vesicles ranging from 70 to 150 nm and have an endocytic origin [172]. In the last decade, numerous in vitro and in vivo studies have employed EVs derived from MSCs to study their effect on different diseases [169,175]. The absence of immunoreactivity and tumorigenicity [175], as well as their stability and specific natural target system, make the use of EVs likely to be safer than cell-based therapies; therefore, they constitute a promising strategy for regenerative medicine purposes [168]. Nonetheless, due to the novelty of this approach, it is still necessary to standardize the currently available protocols and to achieve a better understanding of the mechanisms that underlie their regenerative properties. In this sense, a set of guidelines with minimum criteria for developing studies involving the use of EVs was recently developed by the International Society for EVs [176,177].

Few studies have proposed the therapeutic effect of exosomes isolated from iPSCs on musculoskeletal diseases. For OA treatment, the effects of exosomes obtained from iPSC-MSCs and from synovial MSCs were compared [41]. Exosomes were injected into a mouse OA model and attenuation of the OA was observed using exosomes from both cell types but those from iPSC-MSCs showed better reparative effects after 28 days [41]. Exosomes from iPSC-MSCs were also injected intravenously as treatment in a rat osteonecrosis model [171]. After 21 days, the exosome treatment group showed lower bone loss and enhanced angiogenesis compared with untreated controls. Similar effects were observed in another study using iPSC-MSC exosomes in critical-sized calvarial defects in ovariectomized rats [42].

These results, while encouraging, need further confirmation. Moreover, the mechanisms underlying the reparative effect need to be elucidated for further clinical application.

## 5. Conclusions

The enormous potential expected from iPSCs since they were generated more than a decade ago has been confirmed by recent studies showing their regenerative potential, as well as their ability to recapitulate the characteristics of musculoskeletal diseases in vitro. Nowadays the generation of genetically corrected iPSCs is feasible and that it is possible to identify the cellular and molecular mechanisms underlying musculoskeletal diseases using iPSCs. Despite the many remaining challenges and the long way ahead until iPSC-based therapies become clinical treatments, the use of these cells in the orthopedic field is only expected to increase as our ability to generate more accurate and specific cells improves. The concomitant development of phenotypically and physiologically relevant in vitro models of bone, cartilage, muscle and tendon offers exciting prospects for disease modeling, drug discovery and regenerative medicine applications.

## Figures and Tables

**Table 1:** Summary of disease models of bone, cartilage, nucleus pulposus and muscle disorders generated using iPSCs.

Disease	Suggested Associated Genes	Cell Line Name	Differentiate D Cell Type	Disease-Related Findings	Reference
Marfan’s syndrome (MFS)	FBN1	ISMMSi002-B	-	-	Klein et al. [63]
		MFS-iPSCs	Osteoblasts and vascular smooth muscle cells	Reduced osteogenesis in derivative MSCs, reduced ratio of contracting cells and altered calcium signals in derivative SMCs.	Park et al. [64]
		MFSiPS	Osteoblasts and chondrocytes	Impaired osteogenic differentiation.	Quarto et al. [65]
Andersen’s syndrome (AS)	KCNJ2, KCNJ5	AS-iPS	Osteoblasts	Osteogenic differentiation markers were lower in differentiated AS-iPS compared with control iPSC cells.	Pini et al. [71]
Fibrodysplasia ossificans progressiva (FOP)	ACVR1	FOP iPS	Osteoblasts and chondrocytes	FOP-iPS cells showed a trend towards increased mineralization and enhanced chondrogenesis in vitro.	Matsumoto et al. [72]
			Dermatome, myotome, sclerotome and syndetome cells	OP-iPSC-MSCs showed enhanced chondrogenesis but FOP-iPSC-derived sclerotome did not.	Nakajima et al. [73]
Osteopetrosis	TCIRG1, SNX10, CLCN7, CAII, PLEKHM1, RANKL	BSG-OST14-MSC, ZCD-OST10-MSC, ANC-OST3-MSC	-	-	Okur et al. [40]
		ARO-iPSC1-11	-	-	Xu et al. [74]
		ADO2-iPSCs	-	-	Ou et al. [75]
Osteogenesis imperfecta (OI)	COL1A1	OI-iPSCs	Osteoblasts and osteocytes	Osteogenic marker expression was similar between OI-iPSC-derived cells and healthy ones. However, amounts of calcium deposition of OI-iPSC-derived cells were lower than those of WT-iPSC-derived cells. Also, intracellular Type I collagen was much larger in OI-cells than in healthy cells.	Kawai et al. [76]
		MCRIi018-A, MCRIi018-B	-	-	Howden et al. [77]
		iPSC-OI-FV, iPSC-OI-LV, iPSCe-LV	Osteoblasts	-	Deyle et al. [78]
Turner syndrome (TS)	Monosomy X	TS-iPSC	Osteoblasts and osteoclasts	No differences between osteoblasts from TS-iPSCs and healthy iPSCs but TS-iPSCs showed increased osteoclastogenesis.	Cui et al. [79]
Gaucher disease (GD)	GBA1	GD hiPSC	Osteoblasts	GD iPSC-osteoblasts showed reduced osteogenic differentiation and lysosomal abnormalities that interfered with bone matrix deposition.	Panicker et al. [66]
Li-Fraumeni syndrome (LFS)	TP53, H19	LFS1-A-D, LFS2-A-D, LFS3-A-C	Osteoblasts through MSCs	p53 signaling is active in healthy iPSC-OBs but impaired in LFS. Also, slower and lower osteogenic differentiation in LFS iPSC-MSC with no mineral precipitations observed.	Lee et al. [80]
		LFS iPSCs	Osteoblasts through MSCs	-	Zhou et al. [81]
Familial osteochondritis dissecans (FOCD)	ACAN	FOCD-NS1-iPSC-2/30, FOCD-NS2-iPSC-9/13	Chondrocytes	Chondrocytes from FOCD-NS iPSCs showed glycosaminoglycan (GAG) in the matrix but lack aggrecan, which had a pronounced intracellular localization.	Xu et al. [82]
NOMID arthropathy	NLRP3	NLPR3-iPSCs	Chondrocytes	Chondrocyte-iPSCs produced larger chondrocyte masses than healthy iPSCs owing to GAG overproduction.	Yokoyama et al. [59]
		NLRC4	-	-	Kawasaki et al. [83]
FGFR3-chondrodysplasia	FGFR3	TD1-shFGFR3	Chondrocytes	Small hypertrophic chondrocytes.	Kimura et al. [84]
		TD1-714-3, TD1-10749-1, TD1-315H-2	Chondrocytes	Chondrocyte-iPSCs showed low levels of GAGs in the extracellular matrix (ECM), decreased proliferation and increased apoptosis.	Yamashita et al. [85]
Rheumatoid arthritis	Multifactorial	RA iPSCs	Osteoblasts	-	Lee et al. [86]
Osteoarthritis	Multifactorial	OA iPSCs	Osteoblasts	-	Lee et al. [86]
		N1-FiPS4F#7	-	-	Castro-Viñuelas et al. [87]
		MOA1-FiPS4F#7, MOA2-FiPS4F#17	Chondrocytes	OA iPSC-chondrocytes showed lower levels of collagens and proteoglycans in the ECM than healthy iPSCs.	Castro-Viñuelas et al. [88]
		hSC52/hSC65	Chondrocytes	-	Kim et al. [89]
Myoshi myopathy (MM)	DYSFERLIN	MyoD-hiPSCs	Myocytes	Defective membrane repair in MyoD-MM hiPSC-derived myotubes.	Tanaka et al. [90]
		MM iPS	Myocytes	Dysferlin protein was lower in MM- than in control-iPSC-myocytes.	Kokubu et al. [91]
Limb-girdle muscular dystrophies (LGMD): 1B, 2B, 2A, 2D	Several genes implicated (depending on type)	JF010i-DYSFHZ1	Muscle progenitors	Low levels of dysferlin protein and absence of alpha-sarcoglycan protein were observed in the differentiated LGMD2B- and LGMD2D- iPSCs.	Turan et al. [92]
		9015, 0826 and 0989	Myotubes	LGMD2A-iPSC-myotubes lacked expression of the Calpain 3 protein.	Selvaraj et al. [93]
		R249W	Myogenic cells	Higher proportion of abnormal myotube nuclei compared to control. iPSC myotube nuclei showed morphological defects in nuclear contour ratio.	Steele-Stallard et al. [94]
Duchenne muscular dystrophy (DMD)	Dystrophin	GM05112-M5.1	Myoblasts	Ectopic expression of MyoD in DMD iPSCs led to increased expression levels of Dystrophin but transcripts were truncated.	Abujarour et al. [95]
		DMD-hiPSC-GM05169	Myoblasts	DMD-iPSC-myoblasts showed aberrant expression of inflammation genes and collagens, BMP/TGFβ signaling and reduced myotube formation compared with control iPSCs.	Choi et al. [96]
		UCLi011-A, UCLi012-A	-	-	Ferrari et al. [97]
		ΔEx8-9 iDMD iPSCs	-	-	Kyrychenko et al. [98]
Myotonic dystrophy 1	DMPK	DM1-1 iPS, DM1-2 iPS	Myogenic lineage	iPSC-myogenic progenitors and -myotubes showed intranuclear RNA foci and sequestration of Muscleblind-like protein 1.	Mondragón-González et al. [99]
		DM1-iPSCs	Myogenic lineage	Ribonuclear foci in the undifferentiated and myo-iPSCs but not in healthy ones.	Dastidar et al. [100]
Emery-Dreifuss/LMNA-related congenital muscular dystrophy/dilated cardiomyopathy	LMNA	K32del, L35P	Myogenic cells	LMNA mutant iPSC-myogenic cell produced a higher proportion of abnormal myotube nuclei compared with control.	Steele-Stallard et al. [94]
		DCM	-	-	Ho et al. [101]
		R225X	-	-	Siu et al. [102]
Carnitine palmitotransferase II deficiency	CPT2	CPTIID-iPSC	Myocytes	iPSC-myocytes accumulated more palmitoylcarnitine than control ones.	Yasuno et al. [103]
Valosin-containing protein disease	VCP	VCP-iPSC	Myogenic progenitor cells	Accumulation of autophagy markers in differentiated myogenic-iPSCs.	Llewelyn et al. [104]
		R155C, R191Q	-	-	Ludtmann et al. [105]
Pompe disease	GAA	Pom2 iPSC	Myocytes	iPSC- myocytes showed lysosomal glycogen accumulation and impaired mTORC1 activity.	Yoshida et al. [106]
		GM20124, GM11661	-	-	Higuchi et al. [107]
		GM20089, GM20123, GM04912	-	-	Raval et al. [108]
		PomD-iPSCs	-	-	Huang et al. [109]
Degenerative disc disease	Multifactorial	DDD NP-derived iPSCs	Nucleus pulposus cells	-	Zhu et al. [49]

**Table 2:** Summary of musculoskeletal preclinical repair models using iPSCs.

Disease or Damaged Tissue	Cell Type Used	Animal	Application	Outcome	References
MIA-induced OA rat model	iPSCs and chondro-differentiated iPSCs	Rats	Injected as cell suspension	Histologically, gradual engraftment, improvement of subchondral integrity and articular cartilage matrix production. Better outcome using chondro-differentiated cells.	Zhu et al. [136]
Osteochondral defect model	Mesoderm-differentiated iPSCs	Minipigs	Cells embedded in collagen hydrogel and seeded on bTCP/PLLA scaffolds	Histologically, cartilage formation partially observed at the transplantation sites.	Uto et al. [137]
Osteochondral defect model	iPSC-MSCs and chondro-derived iPSCs	Rats	Pellets on PEG and CS methacrylate scaffolds	hiPSC-derived MSC implants had started to produce a chondrogenic matrix but chondro-derived cells showed stronger GAG and collagen type II staining.	Nejadnik et al. [138]
Osteochondral defect model	iPSC-MSCs	Rabbits	Cells seeded on Matrigel-coated PLGA scaffolds	Better histological quality of in vivo cartilage defect repair in the experimental group compared with controls.	Xu et al. [139]
Osteochondral defect model	Chondrogenic-iPSCs constructs or micromass	Rats	Cell pellets or alginate-hiPSCs constructs	Histologically, hiPSCs showed significantly better quality of cartilage repair than control defects.	Ko et al. [140]
Osteochondral defect model	Cartilaginous tissues derived from iPSCs	Rats and minipigs	Transplantation of hiPSC-derived cartilaginous particles	Histologically, cartilage-like particles were observed to be integrated into native tissue.	Yamashita et al. [60]
Bone defect model	Osteoblasts-differentiated iPSCs	Rats	Cells embedded on PuraMatrix	Good osteogenic properties both in vivo and in vitro. Bone volume and bone mineral content were significantly higher and more newly formed bone than in iPSC-RUNX2 mutated controls.	Saito et al. [141]
Critical size-defect bone model	iPSC-MSCs	Minipigs	Cells seeded on calcium phosphate granules	New bone formation with good osseous consolidation in the central and cortical defect zones but less successful than in the autograft group.	Jungbluth et al. [142]
Dystrophy-induced mice model	Myogenic like cells derived from iPSCs	Mice	Injection	MB1-MyoD-hiPSCs were highly fused with host muscle fibers compared with MB1- MyoD-ESCs and control myoblasts.	Goudenege et al. [143]
Dystrophy-induced mice model	Myogenic like progenitor cells derived from iPSCs	Mice	Injection	Pax7-induced iPS-derived myogenic progenitors resulted in extensive engraftment and improved contractility of muscles.	Darabi et al. [144]
Anterior cruciate ligament injury model	Ligament and osteogenic derivation of iPSCs	Swine	Leeds-Keio constructs	New ACL-like tissue showed morphological and biochemical characteristics resembling those of normal ACL.	Kouroupis et al. [145]
Degenerative disc disease	iPSC-nucleus pulposus cells	Rat	Injection of cells embedded on gelatin microspheres	Histology and imaging results indicated partial restoration of iPSC-nucleus pulposus cells and their ECM and disc height and water content increased.	Xia et al. [48]
Degenerative disc disease	GDF5+ iPSC	Rat	Injection of cells embedded on a thermosensitive hydrogel	Disc height and histology improved after treatment with GDF5+hiPSCs on hydrogel.	Hu et al. [146]
Degenerative disc disease	iPSC-notochordal cells	Pig	Injection of cells resuspended in Geltrex	Good notochordal cell phenotype in vitro and in vivo. Proper functionality of notochordal cells protecting from degeneration and changes in pH level.	Sheyn et al. [147]

## Abbreviations

AS	Andersen’s Syndrome
CRISPR/Cas9	clustered regularly interspaced short palindromic repeats/CRISPR-associated protein 9
CS	chondroitin sulfate
DCM	Dilated cardiomyopathy
DMD	Duchenne Muscular Dystrophy
EB	embryoid body
ECM	extracellular matrix
EDMD	Emery-Dreifuss muscular dystrophy
ESCs	embryonic stem cells
EVs	extracellular vescicles
*FBN1*	FIBRILLIN-1 gene
FOCD	Familial Osteochondritis Disecans
FOP	Fibrodysplasia Ossificans Progressiva
GAGs	glycosaminoglycans
GD	Gaucher Disease
HA	hydroxyapatite
HLA	human leukocyte antigen
IGF-1	Insulin Growth Factor 1
iPSCs	induced pluripotent stem cells
Klf4	Krüppel-like factor
L-CMD	LMNA-related congenital muscular dystrophy
LFS	Li-Fraumeni Syndrome
LGMD	limb-girdle muscular dystrophy
MM	Myoshi Myopathy
MFS	Marfan’s Syndrome
MSCs	mesenchymal stromal cells
*NALP3*	NACHT, LRR and PYD domains-containing protein 3 gene
NOMID	neonatal-onset multisystem inflammatory disease
OA	osteoarthritis
Oct3/4	octamer binding protein 3/4
OI	Osteogenesis Imperfecta
PLGA	poly lactic-co-glycolide
PLLA	poly-L-lactic acid
PEG	Polyethylene Glycol
RA	Rheumatoid Arthritis
SMA	Spinal Muscular Atrophy
Sox2	SRY (sex determining region Y)-box 2
TALENS	transcription activator-like effector nucleases
TCP	beta tricalcium phosphate
ZFNs	zinc finger nucleases

## References

[1] Barruet E., Hsiao E.C. (2016). Using Human Induced Pluripotent Stem Cells to Model Skeletal Diseases. Methods Mol. Biol..

[2] Briggs A.M., Cross M.J., Hoy D.G., Sànchez-Riera L., Blyth F.M., Woolf A.D., March L. (2016). Musculoskeletal Health Conditions Represent a Global Threat to Healthy Aging: A Report for the 2015 World Health Organization World Report on Ageing and Health. Gerontologist.

[3] Li W.-J., Jiao H., Walczak B.E. (2019). Emerging Opportunities for Induced Pluripotent Stem Cells in Orthopaedics. J. Orthop. Transl..

[4] Schofield D.J., Kelly S.J., Shrestha R.N., Callander E., Passey M.E., Percival R. (2012). The Impact of Back Problems on Retirement Wealth. Pain.

[5] Ortiz-Vitali J., Darabi R. (2019). IPSCs As a Platform for Disease Modeling, Drug Screening, and Personalized Therapy in Muscular Dystrophies. Cells.

[6] Ricci F., Vacchetti M., Brusa C., Vercelli L., Davico C., Vitiello B., Mongini T. (2019). New Pharmacotherapies for Genetic Neuromuscular Disorders: Opportunities and Challenges. Expert Rev. Clin. Pharmacol..

[7] Bannuru R.R., Osani M., Vaysbrot E., Arden N., Bennell K., Bierma-Zeinstra S., Kraus V., Lohmander L., Abbott J., Bhandari M. (2019). OARSI Guidelines for the Non-Surgical Management of Knee, Hip, and Polyarticular Osteoarthritis. Osteoarthr. Cartil..

[8] National Research Council (1989). Opportunities in Biology.

[9] Thysen S., Luyten F.P., Lories R.J. (2015). Targets, Models and Challenges in Osteoarthritis Research. Dis. Model. Mech..

[10] Passier R., Orlova V.V., Mummery C. (2016). Complex Tissue and Disease Modeling Using HiPSCs. Cell Stem Cell.

[11] Liu H., Yang L., Yu F.F., Wang S., Wu C., Qu C., Lammi M.J., Guo X. (2017). The Potential of Induced Pluripotent Stem Cells as a Tool to Study Skeletal Dysplasias and Cartilage-Related Pathologic Conditions. Osteoarthr. Cartil..

[12] Singh V.K., Kalsan M., Kumar N., Saini A., Chandra R. (2015). Induced Pluripotent Stem Cells: Applications in Regenerative Medicine, Disease Modeling, and Drug Discovery. Front. Cell Dev. Boil..

[13] Desnuelle C., Dib M., Garrel C., Favier A. (2001). A Double-Blind, Placebo-Controlled Randomized Clinical Trial of α-Tocopherol (vitamin E) in the Treatment of Amyotrophic Lateral Sclerosis. Amyotroph. Lateral Scler..

[14] Shefner J.M., Cudkowicz M.E., Schoenfeld D., Conrad T., Taft J., Chilton M., Urbinelli L., Qureshi M., Zhang H., Pestronk A. (2004). A Clinical Trial of Creatine in ALS. Neurology.

[15] Kumar S., Blangero J., Curran J.E. (2018). Induced Pluripotent Stem Cells in Disease Modeling and Gene Identification. Methods Mol. Biol..

[16] Piñeiro-Ramil M., Sanjurjo-Rodríguez C., Castro-Viñuelas R., Rodríguez-Fernández S., Fuentes-Boquete I., Blanco F., Díaz-Prado S. (2019). Usefulness of Mesenchymal Cell Lines for Bone and Cartilage Regeneration Research. Int. J. Mol. Sci..

[17] Abbott J., Holtzer H. (1966). The Loss of Phenotypic Traits by Differentiated Cells. 3. The Reversible Behavior of Chondrocytes in Primary Cultures. J. Cell Biol..

[18] Holtzer H., Abbott J., Lash J., Holtzer S. (1960). The loss of phenotypic traits by differentiated cells in vitro, I. Dedifferentiation of cartilage cells. Proc. Natl. Acad. Sci. USA.

[19] Khodabukus A., Prabhu N., Wang J., Bursac N. (2018). In Vitro Tissue-Engineered Skeletal Muscle Models for Studying Muscle Physiology and Disease. Adv. Health Mater..

[20] Allen D.D., Caviedes R., Cardenas A.M., Shimahara T., Segura-Aguilar J., Caviedes P.A. (2005). Cell Lines As In Vitro Models for Drug Screening and Toxicity Studies. Drug Dev. Ind. Pharm..

[21] Ebert A.D., Yu J., Rose F.F., Mattis V.B., Lorson C.L., Thomson J.A., Svendsen C.N. (2008). Induced Pluripotent Stem Cells from a Spinal Muscular Atrophy Patient. Nature.

[22] Piñeiro-Ramil M., Castro-Viñuelas R., Sanjurjo-Rodríguez C., Rodríguez-Fernández S., Hermida-Gómez T., Blanco F.J., Fuentes-Boquete I., Díaz-Prado S. (2020). Immortalizing Mesenchymal Stromal Cells from Aged Donors While Keeping Their Essential Features. Stem Cells Int..

[23] Mamchaoui K., Trollet C., Bigot A., Negroni E., Chaouch S., Wolff A., Kandalla P.K., Marie S., Di Santo J.P., Guily J.L.S. (2011). Immortalized Pathological Human Myoblasts: Towards a Universal Tool for the Study of Neuromuscular Disorders. Skelet. Muscle.

[24] Kartsogiannis V., Ng K.W. (2004). Cell Lines and Primary Cell Cultures in the Study of Bone Cell Biology. Mol. Cell. Endocrinol..

[25] Horvath P., Aulner N., Bickle M., Davies A.M., Del Nery E., Ebner D.V., Montoya M.C., Ostling P., Pietiäinen V., Price L.S. (2016). Screening Out Irrelevant Cell-Based Models of Disease. Nat. Rev. Drug Discov..

[26] Akpancar S., Tatar O., Turgut H., Akyildiz F., Ekinci S. (2016). The Current Perspectives of Stem Cell Therapy in Orthopedic Surgery. Arch. Trauma Res..

[27] Castro-Viñuelas R., Sanjurjo-Rodríguez C., Piñeiro-Ramil M., Hermida-Gómez T., Fuentes-Boquete I., De Toro-Santos F., Blanco-García F., Díaz-Prado S. (2018). Induced Pluripotent Stem Cells for Cartilage Repair: Current Status and Future Perspectives. Eur. Cells Mater..

[28] Diederichs S., Klampfleuthner F.A.M., Moradi B., Richter W. (2019). Chondral Differentiation of Induced Pluripotent Stem Cells Without Progression into the Endochondral Pathway. Front. Cell Dev. Boil..

[29] Diekman B.O., Christoforou N., Willard V.P., Sun H., Sanchez-Adams J., Leong K.W., Guilak F. (2012). Cartilage Tissue Engineering Using Differentiated and Purified Induced Pluripotent Stem Cells. Proc. Natl. Acad. Sci. USA.

[30] Jevons L.A., Houghton F.D., Tare R. (2018). Augmentation of Musculoskeletal Regeneration: Role for Pluripotent Stem Cells. Regen. Med..

[31] Kouroupis D., Sanjurjo-Rodriguez C., Jones E., Correa D. (2019). Mesenchymal Stem Cell Functionalization for Enhanced Therapeutic Applications. Tissue Eng. Part B Rev..

[32] Takahashi K., Tanabe K., Ohnuki M., Narita M., Ichisaka T., Tomoda K., Yamanaka S. (2007). Induction of Pluripotent Stem Cells from Adult Human Fibroblasts by Defined Factors. Cell.

[33] Takahashi K., Yamanaka S. (2006). Induction of Pluripotent Stem Cells from Mouse Embryonic and Adult Fibroblast Cultures by Defined Factors. Cell.

[34] Shi Y., Inoue H., Wu J.C., Yamanaka S. (2016). Induced Pluripotent Stem Cell Technology: A Decade of Progress. Nat. Rev. Drug Discov..

[35] Rowe R.G., Daley G.Q. (2019). Induced Pluripotent Stem Cells in Disease Modelling and Drug Discovery. Nat. Rev. Genet..

[36] Doss M.X., Sachinidis A. (2019). Current Challenges of IPSC-Based Disease Modeling and Therapeutic Implications. Cells.

[37] Scudellari M. (2016). How IPS Cells Changed the World. Nature.

[38] Zhao C., Ikeya M. (2018). Generation and Applications of Induced Pluripotent Stem Cell-Derived Mesenchymal Stem Cells. Stem Cells Int..

[39] Dayem A.A., Bin Lee S., Kim K., Lim K.M., Jeon T.-I., Seok J., Cho S.-G., Cho A.S.-G. (2019). Production of Mesenchymal Stem Cells Through Stem Cell Reprogramming. Int. J. Mol. Sci..

[40] Okur F., Cevher I., Özdemir C., Kocaefe Y.C., Uckan-Cetinkaya D. (2019). Osteopetrotic Induced Pluripotent Stem Cells Derived from Patients with Different Disease-Associated Mutations by Non-Integrating Reprogramming Methods. Stem Cell Res. Ther..

[41] Zhu Y., Wang Y., Zhao B., Niu X., Hu B., Li Q., Zhang J., Ding J., Chen Y., Wang Y. (2017). Comparison of Exosomes Secreted by Induced Pluripotent Stem Cell-Derived Mesenchymal Stem Cells and Synovial Membrane-Derived Mesenchymal Stem Cells for the Treatment of Osteoarthritis. Stem Cell Res. Ther..

[42] Qi X., Zhang J., Yuan H., Xu Z., Li Q., Niu X., Hu B., Wang Y., Li X. (2016). Exosomes Secreted by Human-Induced Pluripotent Stem Cell-Derived Mesenchymal Stem Cells Repair Critical-Sized Bone Defects through Enhanced Angiogenesis and Osteogenesis in Osteoporotic Rats. Int. J. Boil. Sci..

[43] Csobonyeiova M., Polak S., Zamborsky R., Danisovic L. (2017). IPS Cell Technologies and Their Prospect for Bone Regeneration and Disease Modeling: A Mini Review. J. Adv. Res..

[44] Wu Q., Yang B., Hu K., Cao C., Man Y., Wang P. (2017). Deriving Osteogenic Cells from Induced Pluripotent Stem Cells for Bone Tissue Engineering. Tissue Eng. Part B Rev..

[45] Perez J.R., Kouroupis D., Li D.J., Best T.M., Kaplan L., Correa D. (2018). Tissue Engineering and Cell-Based Therapies for Fractures and Bone Defects. Front. Bioeng. Biotechnol..

[46] Driessen B.J., Logie C., Vonk L. (2017). Cellular Reprogramming for Clinical Cartilage Repair. Cell Boil. Toxicol..

[47] Tang R., Jing L., Willard V.P., Wu C.-L., Guilak F., Chen J., Setton L.A. (2018). Differentiation of Human Induced Pluripotent Stem Cells into Nucleus Pulposus-Like Cells. Stem Cell Res. Ther..

[48] Xia K., Zhu J., Hua J., Gong Z., Yu C., Zhou X., Wang J., Huang X., Yu W., Li L. (2019). Intradiscal Injection of Induced Pluripotent Stem Cell-Derived Nucleus Pulposus-Like Cell-Seeded Polymeric Microspheres Promotes Rat Disc Regeneration. Stem Cells Int..

[49] Zhu Y.-X., Liang Y., Zhu H., Lian C., Wang L., Wang Y., Gu H., Zhou G., Yu X. (2017). The Generation and Functional Characterization of Induced Pluripotent Stem Cells from Human Intervertebral Disc Nucleus Pulposus Cells. Oncotarget.

[50] De Oñate L., Garreta E., Tarantino C., Martínez E., Capilla E., Navarro I., Gutiérrez J., Samitier J., Campistol J.M., Muñoz-Cánovas P. (2015). Research on Skeletal Muscle Diseases Using Pluripotent Stem Cells. Muscle Cell and Tissue.

[51] Avior Y., Sagi I., Benvenisty N. (2016). Pluripotent Stem Cells in Disease Modelling and Drug Discovery. Nat. Rev. Mol. Cell Boil..

[52] Huang C.-Y., Liu C.-L., Ting C.-Y., Chiu Y.-T., Cheng Y.-C., Nicholson M.W., Hsieh P.C. (2019). Human IPSC Banking: Barriers and Opportunities. J. Biomed. Sci..

[53] Park I.-H., Arora N., Huo H., Maherali N., Ahfeldt T., Shimamura A., Lensch W., Cowan C., Hochedlinger K., Daley G.Q. (2008). Disease-Specific Induced Pluripotent Stem Cells. Cell.

[54] Tashiro K., Inamura M., Kawabata K., Sakurai F., Yamanishi K., Hayakawa T., Mizuguchi H. (2009). Efficient Adipocyte and Osteoblast Differentiation from Mouse Induced Pluripotent Stem Cells by Adenoviral Transduction. Stem Cells.

[55] Kao C.-L., Tai L.-K., Chiou S.-H., Chen Y.-J., Lee K.-H., Chou S.-J., Chang Y.-L., Chang C.-M., Chen S.-J., Ku H.-H. (2010). Resveratrol Promotes Osteogenic Differentiation and Protects Against Dexamethasone Damage in Murine Induced Pluripotent Stem Cells. Stem Cells Dev..

[56] Ardeshirylajimi A., Soleimani M. (2015). Enhanced Growth and Osteogenic Differentiation of Induced Pluripotent Stem Cells by Extremely Low-Frequency Electromagnetic Field. Cell. Mol. Biol..

[57] Umeda K., Zhao J., Simmons P., Stanley E., Elefanty A., Nakayama N. (2012). Human Chondrogenic Paraxial Mesoderm, Directed Specification and Prospective Isolation from Pluripotent Stem Cells. Sci. Rep..

[58] Boreström C., Simonsson S., Enochson L., Bigdeli N., Brantsing C., Ellerström C., Hyllner J., Lindahl A. (2014). Footprint-Free Human Induced Pluripotent Stem Cells from Articular Cartilage with Redifferentiation Capacity: A First Step Toward a Clinical-Grade Cell Source. Stem Cells Transl. Med..

[59] Yokoyama K., Ikeya M., Umeda K., Oda H., Nodomi S., Nasu A., Matsumoto Y., Izawa K., Horigome K., Kusaka T. (2014). Enhanced Chondrogenesis of Induced Pluripotent Stem Cells from Patients with Neonatal-Onset Multisystem Inflammatory Disease Occurs via the Caspase 1-Independent cAMP/Protein Kinase A/CREB Pathway. Arthritis Rheumatol..

[60] Yamashita A., Morioka M., Yahara Y., Okada M., Kobayashi T., Kuriyama S., Matsuda S., Tsumaki N. (2015). Generation of Scaffoldless Hyaline Cartilaginous Tissue from Human IPSCs. Stem Cell Rep..

[61] Chal J., Oginuma M., Al Tanoury Z., Gobert B., Sumara O., Hick A., Bousson F., Zidouni Y., Mursch C., Moncuquet P. (2015). Differentiation of Pluripotent Stem Cells to Muscle Fiber to Model Duchenne Muscular Dystrophy. Nat. Biotechnol..

[62] Zhang C., Yuan H., Liu H., Chen X., Lu P., Zhu T., Yang L., Yin Z., Heng B.C., Zhang Y. (2015). Well-Aligned Chitosan-Based Ultrafine Fibers Committed Teno-Lineage Differentiation of Human Induced Pluripotent Stem Cells for Achilles Tendon Regeneration. Biomaterials.

[63] Klein S., Dvornik J.L., Yarrabothula A.R., Schaniel C. (2017). A Marfan Syndrome Human Induced Pluripotent Stem Cell Line with a Heterozygous *FBN1* c.4082G > A Mutation, ISMMSi002-B, for Disease Modeling. Stem Cell Res..

[64] Park J.W., Yan L., Stoddard C., Wang X., Yue Z., Crandall L., Robinson T., Chang Y., Denton K., Li E. (2017). Recapitulating and Correcting Marfan Syndrome in a Cellular Model. Int. J. Boil. Sci..

[65] Quarto N.P., Leonard B., Li S., Marchand M., Anderson E., Behr B., Francke U., Pera R.A.R., Chiao E., Longaker M.T. (2011). Skeletogenic Phenotype of Human Marfan Embryonic Stem Cells Faithfully Phenocopied by Patient-Specific Induced-Pluripotent Stem Cells. Proc. Natl. Acad. Sci. USA.

[66] Panicker L.M., Srikanth M.P., Castro-Gomes T., Miller D., Andrews N.W., Feldman R.A. (2018). Gaucher Disease IPSC-Derived Osteoblasts Have Developmental and Lysosomal Defects That Impair Bone Matrix Deposition. Hum. Mol. Genet..

[67] Cai B., Sun S., Li Z., Zhang X., Ke Y., Yang J., Li X. (2018). Application of CRISPR/Cas9 Technologies Combined with IPSCs in the Study and Treatment of Retinal Degenerative Diseases. Qual. Life Res..

[68] Ben Jehuda R., Shemer Y., Binah O. (2018). Genome Editing in Induced Pluripotent Stem Cells Using CRISPR/Cas9. Stem Cell Rev. Rep..

[69] Chuang K., Fields M.A., Del Priore L.V. (2017). Potential of Gene Editing and Induced Pluripotent Stem Cells (iPSCs) in Treatment of Retinal Diseases. Yale J. Biol. Med..

[70] Seah Y.F.S., El Farran C., Warrier T., Xu J., Loh Y.-H. (2015). Induced Pluripotency and Gene Editing in Disease Modelling: Perspectives and Challenges. Int. J. Mol. Sci..

[71] Pini J., Rouleau M., Desnuelle C., Sacconi S., Bendahhou S. (2016). Modeling Andersen’s Syndrome in Human Induced Pluripotent Stem Cells. Stem Cells Dev..

[72] Matsumoto Y., Hayashi Y., Schlieve C.R., Ikeya M., Kim H., Nguyen T.D., Sami S., Baba S., Barruet E., Nasu A. (2013). Induced Pluripotent Stem Cells from Patients with Human Fibrodysplasia Ossificans Progressiva Show Increased Mineralization and Cartilage Formation. Orphanet J. Rare Dis..

[73] Nakajima T., Shibata M., Nishio M., Nagata S., Alev C., Sakurai H., Toguchida J., Ikeya M. (2018). Modeling Human Somite Development and Fibrodysplasia Ossificans Progressiva with Induced Pluripotent Stem Cells. Development.

[74] Xu M., Stattin E.-L., Murphy M., Barry F.P. (2017). Generation of Induced Pluripotent Stem Cells (ARO-IPSC1-11) from a Patient with Autosomal Recessive Osteopetrosis Harboring the c.212 + 1G > T Mutation in *SNX10* Gene. Stem Cell Res..

[75] Ou M., Li C., Tang D., Xue W., Xu Y., Zhu P., Li B., Xie J., Chen J., Sui W. (2019). Genotyping, Generation and Proteomic Profiling of the First Human Autosomal Dominant Osteopetrosis Type II-Specific Induced Pluripotent Stem Cells. Stem Cell Res. Ther..

[76] Kawai S., Yoshitomi H., Sunaga J., Alev C., Nagata S., Nishio M., Hada M., Koyama Y., Uemura M., Sekiguchi K. (2019). In Vitro Bone-Like Nodules Generated from Patient-Derived IPSCs Recapitulate Pathological Bone Phenotypes. Nat. Biomed. Eng..

[77] Howden S., Far H.H., Motazedian A., Elefanty A.G., Stanley E.G., Lamandé S.R., Bateman J.F. (2019). The Use of Simultaneous Reprogramming and Gene Correction to Generate an Osteogenesis Imperfecta Patient *COL1A1* c. 3936 G > T IPSC Line and an Isogenic Control IPSC Line. Stem Cell Res..

[78] Deyle D.R., Khan I.F., Ren G., Wang P.-R., Kho J., Schwarze U., Russell D.W. (2012). Normal Collagen and Bone Production by Gene-Targeted Human Osteogenesis Imperfecta IPSCs. Mol. Ther..

[79] Cui X., Cui Y., Shi L., Luan J., Zhou X., Han J. (2019). A Preliminary Study on the Mechanism of Skeletal Abnormalities in Turner Syndrome Using Inducing Pluripotent Stem Cells (iPS)—Based Disease Models. Intractable Rare Dis. Res..

[80] Lee D.-F., Su J., Kim H.S., Chang B., Papatsenko D., Zhao R., Yuan Y., Gingold J., Xia W., Darr H. (2015). Modeling Familial Cancer with Induced Pluripotent Stem Cells. Cell.

[81] Zhou R.-J., Xu A., Tu J., Liu M., Gingold J., Zhao R., Lee D.-F. (2018). Modeling Osteosarcoma Using Li-Fraumeni Syndrome Patient-Derived Induced Pluripotent Stem Cells. J. Vis. Exp..

[82] Xu M., Stattin E.-L., Shaw G., Heinegård D., Sullivan G.J., Wilmut I., Colman A., Önnerfjord P., Khabut A., Aspberg A. (2016). Chondrocytes Derived from Mesenchymal Stromal Cells and Induced Pluripotent Cells of Patients with Familial Osteochondritis Dissecans Exhibit an Endoplasmic Reticulum Stress Response and Defective Matrix Assembly. Stem Cells Transl. Med..

[83] Kawasaki Y., Oda H., Ito J., Niwa A., Tanaka T., Hijikata A., Seki R., Nagahashi A., Osawa M., Asaka I. (2017). Identification of a High-Frequency Somatic NLRC4 Mutation as a Cause of Autoinflammation by Pluripotent Cell-Based Phenotype Dissection. Arthritis Rheumatol..

[84] Kimura T., Ozaki T., Fujita K., Yamashita A., Morioka M., Ozono K., Tsumaki N. (2018). Proposal of Patient-Specific Growth Plate Cartilage Xenograft Model for FGFR3 Chondrodysplasia. Osteoarthr. Cartil..

[85] Yamashita A., Morioka M., Kishi H., Kimura T., Yahara Y., Okada M., Fujita K., Sawai H., Ikegawa S., Tsumaki N. (2014). Statin Treatment Rescues FGFR3 Skeletal Dysplasia Phenotypes. Nature.

[86] Lee J., Kim Y., Yi H., Diecke S., Kim J., Jung H., Rim Y.A., Jung S.M., Kim M., Kim Y.G. (2014). Generation of Disease-Specific Induced Pluripotent Stem Cells from Patients with Rheumatoid Arthritis and Osteoarthritis. Arthr. Res. Ther..

[87] Castro-Viñuelas R., Sanjurjo-Rodríguez C., Piñeiro-Ramil M., Rodríguez-Fernández S., Fuentes-Boquete I., Blanco F., Díaz-Prado S. (2020). Generation of a Human Control IPS Cell Line (ESi080-A) from a Donor with No Rheumatic Diseases. Stem Cell Res..

[88] Castro-Viñuelas R., Sanjurjo-Rodríguez C., Piñeiro-Ramil M., Hermida-Gómez T., Rodríguez-Fernández S., Oreiro N., De Toro J., Fuentes I., Blanco F.J., Díaz-Prado S. (2020). Generation and Characterization of Human Induced Pluripotent Stem Cells (iPSCs) from Hand Osteoarthritis Patient-Derived Fibroblasts. Sci. Rep..

[89] Kim M.-J., Son M.J., Son M.-Y., Seol B., Kim J., Park J., Kim J.H., Kim Y.-H., Park S.A., Lee C. (2011). Generation of Human Induced Pluripotent Stem Cells from Osteoarthritis Patient-Derived Synovial Cells. Arthritis Rheum..

[90] Tanaka A., Woltjen K., Miyake K., Hotta A., Ikeya M., Yamamoto T., Nishino T., Shoji E., Sehara-Fujisawa A., Manabe Y. (2013). Efficient and Reproducible Myogenic Differentiation from Human IPS Cells: Prospects for Modeling Miyoshi Myopathy in Vitro. PLoS ONE.

[91] Kokubu Y., Nagino T., Sasa K., Oikawa T., Miyake K., Kume A., Fukuda M., Fuse H., Tozawa R., Sakurai H. (2019). Phenotypic Drug Screening for Dysferlinopathy Using Patient-Derived Induced Pluripotent Stem Cells. Stem Cells Transl. Med..

[92] Turan S., Farruggio A.P., Srifa W., Day J.W., Calos M.P. (2016). Precise Correction of Disease Mutations in Induced Pluripotent Stem Cells Derived from Patients with Limb Girdle Muscular Dystrophy. Mol. Ther..

[93] Selvaraj S., Dhoke N.R., Kiley J., Mateos-Aierdi A.J., Tungtur S., Mondragon-Gonzalez R., Killeen G., Oliveira V.K., De Munain A.L., Perlingeiro R.C. (2019). Gene Correction of LGMD2A Patient-Specific IPSCs for the Development of Targeted Autologous Cell Therapy. Mol. Ther..

[94] Steele-Stallard H.B., Pinton L., Sarcar S., Ozdemir T., Maffioletti S.M., Zammit P.S., Tedesco F.S. (2018). Modeling Skeletal Muscle Laminopathies Using Human Induced Pluripotent Stem Cells Carrying Pathogenic LMNA Mutations. Front. Physiol..

[95] Abujarour R., Bennett M., Valamehr B., Lee T.T., Robinson M., Robbins D., Le T., Lai K., Flynn P. (2014). Myogenic Differentiation of Muscular Dystrophy-Specific Induced Pluripotent Stem Cells for Use in Drug Discovery. Stem Cells Transl. Med..

[96] Choi I.Y., Lim H., Estrellas K., Mula J., Cohen T.V., Zhang Y., Donnelly C.J., Richard J.-P., Kim Y.J., Kim H. (2016). Concordant But Varied Phenotypes Among Duchenne Muscular Dystrophy Patient-Specific Myoblasts Derived Using a Human IPSC-Based Model. Cell Rep..

[97] Ferrari G., Muntoni F., Tedesco F.S. (2020). Generation of Two Genomic-Integration-Free DMD IPSC Lines with Mutations Affecting All Dystrophin Isoforms and Potentially Amenable to Exon-Skipping. Stem Cell Res..

[98] Kyrychenko V., Kyrychenko S., Tiburcy M., Shelton J.M., Long C., Schneider J.W., Zimmermann W.-H., Bassel-Duby R., Olson E.N. (2017). Functional Correction of Dystrophin Actin Binding Domain Mutations by Genome Editing. JCI Insight.

[99] Modragón-González R., Perlingeiro R.C.R. (2018). Recapitulating Muscle Disease Phenotypes with Myotonic Dystrophy 1 Induced Pluripotent Stem Cells: A Tool for Disease Modeling and Drug Discovery. Dis. Model. Mech..

[100] Dastidar S., Ardui S., Singh K., Majumdar D., Nair N., Fu Y., Reyon D., Samara E., Gerli M.F.M., Klein A.F. (2018). Efficient CRISPR/Cas9-Mediated Editing of Trinucleotide Repeat Expansion in Myotonic Dystrophy Patient-Derived IPS and Myogenic Cells. Nucleic Acids Res..

[101] Ho J.C.Y., Zhou T., Lai W.-H., Huang Y., Chan Y.-C., Li X., Wong N.L.Y., Li Y., Au K.-W., Guo D. (2011). Generation of Induced Pluripotent Stem Cell Lines from 3 Distinct Laminopathies Bearing Heterogeneous Mutations in Lamin A/C. Aging.

[102] Siu C.-W., Lee Y.-K., Ho J.C.-Y., Lai W.-H., Chan Y.-C., Ng K.-M., Wong L.-Y., Au K.-W., Lau Y.-M., Zhang J. (2012). Modeling of Lamin A/C Mutation Premature Cardiac Aging Using Patient-Specific Induced Pluripotent Stem Cells. Aging.

[103] Yasuno T., Osafune K., Sakurai H., Asaka I., Tanaka A., Yamaguchi S., Yamada K., Hitomi H., Arai S., Kurose Y. (2014). Functional Analysis of IPSC-Derived Myocytes from a Patient with Carnitine Palmitoyltransferase II Deficiency. Biochem. Biophys. Res. Commun..

[104] Llewellyn K.J., Nalbandian A., Weiss L.N., Chang I., Yu H., Khatib B., Tan B., Scarfone V., Kimonis V. (2017). Myogenic Differentiation of VCP Disease-Induced Pluripotent Stem Cells: A Novel Platform for Drug Discovery. PLoS ONE.

[105] Ludtmann M.H.R., Arber C.E., Bartolome F., De Vicente M., Preza E., Carro E., Houlden H., Gandhi S., Wray S., Abramov A.Y. (2017). Mutations in Valosin-Containing Protein (VCP) Decrease ADP/ATP Translocation across the Mitochondrial Membrane and Impair Energy Metabolism in Human Neurons. J. Boil. Chem..

[106] Yoshida T., Awaya T., Jonouchi T., Kimura R., Kimura S., Era T., Heike T., Sakurai H. (2017). A Skeletal Muscle Model of Infantile-Onset Pompe Disease with Patient-Specific IPS Cells. Sci. Rep..

[107] Higuchi T., Kawagoe S., Otsud M., Shimad Y., Kobayashi H., Hirayama R., Eto K., Ida H., Ohashi T., Nakauchi H. (2014). The Generation of Induced Pluripotent Stem Cells (iPSCs) from Patients with Infantile and Late-Onset Types of Pompe Disease and the Effects of Treatment with Acid-α-Glucosidase in Pompe’s IPSCs. Mol. Genet. Metab..

[108] Raval K.K., Tao R., White B.E., De Lange W.J., Koonce C.H., Yu J., Kishnani P.S., Thomson J.A., Mosher D.F., Ralphe J.C. (2014). Pompe Disease Results in a Golgi-Based Glycosylation Deficit in Human Induced Pluripotent Stem Cell-Derived Cardiomyocytes. J. Boil. Chem..

[109] Huang H.-P., Chen H.F., Chuang C.-Y., Stone L., Li L.-T., Ho H.-N., Hwu W., Chien C., Chiang S.-C., Kuo H.-C. (2011). Human Pompe Disease-Induced Pluripotent Stem Cells for Pathogenesis Modeling, Drug Testing and Disease Marker Identification. Hum. Mol. Genet..

[110] Mikami Y., Matsumoto T., Kano K., Toriumi T., Somei M., Honda M.J., Komiyama K. (2013). Current Status of Drug Therapies for Osteoporosis and the Search for Stem Cells Adapted for Bone Regenerative Medicine. Anat. Sci. Int..

[111] Nakajima T., Ikeya M. (2019). Insights into the Biology of Fibrodysplasia Ossificans Progressiva Using Patient-Derived Induced Pluripotent Stem Cells. Regen. Ther..

[112] Kuroda Y., Yuasa S., Watanabe Y., Ito S., Egashira T., Seki T., Hattori T., Ohno S., Kodaira M., Suzuki T. (2017). Flecainide Ameliorates Arrhythmogenicity through NCX Flux in Andersen-Tawil Syndrome-IPS Cell-Derived Cardiomyocytes. Biochem. Biophys. Rep..

[113] Awad O., Sarkar C., Panicker L.M., Sgambato J.A., Lipinski M.M., Miller D., Zeng X., Feldman R.A. (2015). Altered TFEB-Mediated Lysosomal Biogenesis in Gaucher Disease IPSC-Derived Neuronal Cells. Hum. Mol. Genet..

[114] Sun Y., Florer J., Mayhew C.N., Jia Z., Zhao Z., Xu K., Ran H., Liou B., Zhang W., Setchell K.D.R. (2015). Properties of Neurons Derived from Induced Pluripotent Stem Cells of Gaucher Disease Type 2 Patient Fibroblasts: Potential Role in Neuropathology. PLoS ONE.

[115] Kodaka Y., Rabu G., Asakura A. (2017). Skeletal Muscle Cell Induction from Pluripotent Stem Cells. Stem Cells Int..

[116] Darabi R., Arpke R.W., Irion S., Dimos J.T., Grskovic M., Kyba M., Perlingeiro R.C.R. (2012). Human ES- and IPS-Derived Myogenic Progenitors Restore DYSTROPHIN and Improve Contractility Upon Transplantation in Dystrophic Mice. Cell Stem Cell.

[117] Xia G., Terada N., Ashizawa T. (2018). Human IPSC Models to Study Orphan Diseases: Muscular Dystrophies. Curr. Stem Cell Rep..

[118] Hagan M., Ashraf M., Kim I.-M., Weintraub N.L., Tang Y. (2018). Effective Regeneration of Dystrophic Muscle Using Autologous IPSC-Derived Progenitors with CRISPR-Cas9 Mediated Precise Correction. Med. Hypotheses.

[119] Maffioletti S.M., Sarcar S., Henderson A.B., Mannhardt I., Pinton L., Moyle L.A., Steele-Stallard H., Cappellari O., Wells K.E., Ferrari G. (2018). Three-Dimensional Human IPSC-Derived Artificial Skeletal Muscles Model Muscular Dystrophies and Enable Multilineage Tissue Engineering. Cell Rep..

[120] Weiler T., Bashir R., Anderson L.V.B., Davison K., Moss J.A., Britton S., Nylen E., Keers S., Vafiadaki E., Greenberg C.R. (1999). Identical Mutation in Patients with Limb Girdle Muscular Dystrophy Type 2B Or Miyoshi Myopathy Suggests a Role for Modifier Gene(s). Hum. Mol. Genet..

[121] Huang H.-P., Chiang W., Stone L., Kang C.-K., Chuang C.-Y., Kuo H.-C. (2019). Using Human Pompe Disease-Induced Pluripotent Stem Cell-Derived Neural Cells to Identify Compounds with Therapeutic Potential. Hum. Mol. Genet..

[122] Mitzelfelt K.A., Limphong P., Choi M.J., Kondrat F.D.L., Lai S., Kolander K.D., Kwok W.-M., Dai Q., Grzybowski M.N., Zhang H. (2016). The Human 343delT HSPB5 Chaperone Associated with Early-Onset Skeletal Myopathy Causes Defects in Protein Solubility. J. Boil. Chem..

[123] Loebel C., Burdick J.A. (2018). Engineering Stem and Stromal Cell Therapies for Musculoskeletal Tissue Repair. Cell Stem Cell.

[124] Sakai D., Andersson G.B.J. (2015). Stem Cell Therapy for Intervertebral Disc Regeneration: Obstacles and Solutions. Nat. Rev. Rheumatol..

[125] Sakai D., Schol J. (2017). Cell Therapy for Intervertebral Disc Repair: Clinical Perspective. J. Orthop. Transl..

[126] Cuthbert R.J., Jones E., Sanjurjo-Rodríguez C., Lotfy A., Ganguly P., Churchman S.M., Castana P., Tan H.B., McGonagle D., Papadimitriou E. (2020). Regulation of Angiogenesis Discriminates Tissue Resident MSCs from Effective and Defective Osteogenic Environments. J. Clin. Med..

[127] Piñeiro-Ramil M., Castro-Viñuelas R., Sanjurjo-Rodríguez C., Hermida-Gómez T., Fuentes-Boquete I., De Toro-Santos F.J., Blanco-García F.J., Díaz-Prado S.M. (2017). Cell Therapy and Tissue Engineering for Cartilage Repair. Cartilage Repair and Regeneration.

[128] Monaco M.L., Merckx G., Ratajczak J., Gervois P., Hilkens P., Clegg P., Bronckaers A., Vandeweerd J.-M., Lambrichts I. (2018). Stem Cells for Cartilage Repair: Preclinical Studies and Insights in Translational Animal Models and Outcome Measures. Stem Cells Int..

[129] Rama P., Matuska S., Paganoni G., Spinelli A., De Luca M., Pellegrini G. (2010). Limbal Stem-Cell Therapy and Long-Term Corneal Regeneration. N. Engl. J. Med..

[130] Duarte R.F., Labopin M., Bader P., Basak G.W., Bonini C., Chabannon C., Corbacioglu S., Dreger P., Dufour C., Gennery A.R. (2019). Indications for Haematopoietic Stem Cell Transplantation for Haematological Diseases, Solid Tumours and Immune Disorders: Current Practice in Europe, 2019. Bone Marrow Transplant..

[131] Ong C.S., Yesantharao P., Huang C.-Y., Mattson G., Boktor J., Fukunishi T., Zhang H., Hibino N. (2017). 3D Bioprinting Using Stem Cells. Pediatr. Res..

[132] Kamei N., Adachi N., Ochi M. (2018). Magnetic Cell Delivery for the Regeneration of Musculoskeletal and Neural Tissues. Regen. Ther..

[133] De Peppo G.M., Marcos-Campos I., Kahler D.J., Alsalman D., Shang L., Vunjak-Novakovic G., Marolt D. (2013). Engineering Bone Tissue Substitutes from Human Induced Pluripotent Stem Cells. Proc. Natl. Acad. Sci. USA.

[134] Hynes K., Menicanin D., Mrozik K.M., Gronthos S., Bartold P.M. (2013). Generation of Functional Mesenchymal Stem Cells from Different Induced Pluripotent Stem Cell Lines. Stem Cells Dev..

[135] Phillips M.D., Kuznetsov S.A., Cherman N., Park K., Chen K.G., McClendon B.N., Hamilton R.S., McKay R.D., Chenoweth J.G., Mallon B.S. (2014). Directed Differentiation of Human Induced Pluripotent Stem Cells Toward Bone and Cartilage: In Vitro Versus In Vivo Assays. Stem Cells Transl. Med..

[136] Zhu Y.-X., Wu X., Liang Y., Gu H., Song K., Zou X., Zhou G.Q. (2016). Repair of Cartilage Defects in Osteoarthritis Rats with Induced Pluripotent Stem Cell Derived Chondrocytes. BMC Biotechnol..

[137] Uto S., Nishizawa S., Hikita A., Takato T., Hoshi K. (2018). Application of Induced Pluripotent Stem Cells for Cartilage Regeneration in CLAWN Miniature Pig Osteochondral Replacement Model. Regen. Ther..

[138] Nejadnik H., Diecke S., Lenkov O.D., Chapelin F., Donig J., Tong X.-M., Derugin N., Chan R.C.F., Gaur A., Yang F. (2015). Improved Approach for Chondrogenic Differentiation of Human Induced Pluripotent Stem Cells. Stem Cell Rev. Rep..

[139] Xu X., Shi D., Liu Y., Yao Y., Dai J., Xu Z., Chen D., Teng H., Jiang Q. (2017). In Vivo Repair of Full-Thickness Cartilage Defect with Human IPSC-Derived Mesenchymal Progenitor Cells in a Rabbit Model. Exp. Ther. Med..

[140] Ko J.-Y., Kim K.-I., Park S., Im G.-I. (2014). In Vitro Chondrogenesis and in Vivo Repair of Osteochondral Defect with Human Induced Pluripotent Stem Cells. Biomaterials.

[141] Saito A., Ooki A., Nakamura T., Onodera S., Hayashi K., Hasegawa D., Okudaira T., Watanabe K., Kato H., Onda T. (2018). Targeted Reversion of Induced Pluripotent Stem Cells from Patients with Human Cleidocranial Dysplasia Improves Bone Regeneration in a Rat Calvarial Bone Defect Model. Stem Cell Res. Ther..

[142] Jungbluth P., Spitzhorn L.-S., Grassmann J., Tanner S., Latz D., Rahman S., Bohndorf M., Wruck W., Sager M., Grotheer V. (2019). Human IPSC-Derived IMSCs Improve Bone Regeneration in Mini-Pigs. Bone Res..

[143] Goudenege S., Lebel C., Huot N.B., Dufour C., Fujii I., Gekas J., Rousseau J., Tremblay J.P. (2012). Myoblasts Derived from Normal HESCs and Dystrophic HiPSCs Efficiently Fuse with Existing Muscle Fibers Following Transplantation. Mol. Ther..

[144] Darabi R., Pan W., Bosnakovski D., Baik J., Kyba M., Perlingeiro R.C.R. (2011). Functional Myogenic Engraftment from Mouse IPS Cells. Stem Cell Rev. Rep..

[145] Kouroupis D., Kyrkou A., Triantafyllidi E., Katsimpoulas M., Chalepakis G., Goussia A., Georgoulis A., Murphy C., Fotsis T. (2016). Generation of Stem Cell-Based Bioartificial Anterior Cruciate Ligament (ACL) Grafts for Effective ACL Rupture Repair. Stem Cell Res..

[146] Hu A., Xing R., Jiang L., Li Z., Liu P., Wang H., Xi-Lei L., Dong J. (2020). Thermosensitive Hydrogels Loaded with human-induced Pluripotent Stem Cells Overexpressing Growth Differentiation factor-5 Ameliorate Intervertebral Disc Degeneration in Rats. J. Biomed. Mater. Res. Part B Appl. Biomater..

[147] Sheyn D., Ben-David S., Tawackoli W., Zhou Z., Salehi K., Bez M., De Mel S., Chan V., Roth J., Avalos P. (2019). Human IPSCs Can Be Differentiated into Notochordal Cells That Reduce Intervertebral Disc Degeneration in a Porcine Model. Theranostics.

[148] Jeon O.H., Panicker L.M., Lu Q., Chae J.J., Feldman R.A., Elisseeff J.H. (2016). Human IPSC-Derived Osteoblasts and Osteoclasts Together Promote Bone Regeneration in 3D Biomaterials. Sci. Rep..

[149] Wrighton K.H. (2017). Stem Cells: The Different Flavours of IPS Cells. Nat. Rev. Genet..

[150] Boland M.J., Nazor K.L., Loring J.F. (2014). Epigenetic Regulation of Pluripotency and Differentiation. Circ. Res..

[151] Kim K., Doi A., Wen B., Ng K., Zhao R., Cahan P., Kim J., Aryee M.J., Ji H., Ehrlich L.I.R. (2010). Epigenetic Memory in Induced Pluripotent Stem Cells. Nature.

[152] Banito A., Gil J. (2010). Induced Pluripotent Stem Cells and Senescence: Learning the Biology to Improve the Technology. EMBO Rep..

[153] Muñoz-Espín D., Cañamero M., Maraver A., Gómez-López G., Contreras J., Murillo-Cuesta S., Rodriguez-Veiga E., Varela-Nieto I., Ruberte J., Collado M. (2013). Programmed Cell Senescence During Mammalian Embryonic Development. Cell.

[154] Tidball A.M., Dang L.T., Glenn T.W., Kilbane E.G., Klarr D.J., Margolis J.L., Uhler M.D., Parent J.M. (2017). Rapid Generation of Human Genetic Loss-of-Function IPSC Lines by Simultaneous Reprogramming and Gene Editing. Stem Cell Rep..

[155] Glicksman M.A. (2018). Induced Pluripotent Stem Cells: The Most Versatile Source for Stem Cell Therapy. Clin. Ther..

[156] Li M., Suzuki K., Kim N.Y., Liu G.-H., Belmonte J.C.I. (2013). A Cut above the Rest: Targeted Genome Editing Technologies in Human Pluripotent Stem Cells. J. Boil. Chem..

[157] Cohen D.E., Melton D. (2011). Turning Straw into Gold: Directing Cell Fate for Regenerative Medicine. Nat. Rev. Genet..

[158] Smith A.S., Macadangdang J., Leung W., Laflamme M., Kim D.-H. (2016). Human IPSC-Derived Cardiomyocytes and Tissue Engineering Strategies for Disease Modeling and Drug Screening. Biotechnol. Adv..

[159] Barreca M.M., Cancemi P., Geraci F. (2020). Mesenchymal and Induced Pluripotent Stem Cells-Derived Extracellular Vesicles: The New Frontier for Regenerative Medicine?. Cells.

[160] Boyd A.S., Rodrigues N.P., Lui K.O., Fu X., Xu Y. (2012). Concise Review: Immune Recognition of Induced Pluripotent Stem Cells. Stem Cells.

[161] Alvarez-Palomo B., Vives J., Casaroli-Marano R.P., Gomez S.G., Rodriguez Gómez L., Edel M.J., Querol Giner S. (2019). Adapting Cord Blood Collection and Banking Standard Operating Procedures for HLA-Homozygous Induced Pluripotent Stem Cells Production and Banking for Clinical Application. J. Clin. Med..

[162] Simonson O.E., Domogatskaya A., Volchkov P., Rodin S. (2015). The Safety of Human Pluripotent Stem Cells in Clinical Treatment. Ann. Med..

[163] Deuse T., Hu X., Gravina A., Wang D., Tediashvili G., De C., Thayer W.O., Wahl A., Garcia J.V., Reichenspurner H. (2019). Hypoimmunogenic Derivatives of Induced Pluripotent Stem Cells Evade Immune Rejection in Fully Immunocompetent Allogeneic Recipients. Nat. Biotechnol..

[164] Uto S., Nishizawa S., Takasawa Y., Asawa Y., Fujihara Y., Takato T., Hoshi K. (2013). Bone and Cartilage Repair by Transplantation of Induced Pluripotent Stem Cells in Murine Joint Defect Model. Biomed. Res..

[165] Lee J., Taylor S.E.B., Smeriglio P., Lai J., Maloney W.J., Yang F., Bhutani N. (2015). Early Induction of a Prechondrogenic Population Allows Efficient Generation of Stable Chondrocytes from Human Induced Pluripotent Stem Cells. FASEB J..

[166] Cunningham J.J., Ulbright T.M., Pera M.F., Looijenga L.H.J. (2012). Lessons from Human Teratomas to Guide Development of Safe Stem Cell Therapies. Nat. Biotechnol..

[167] Kotaka S., Wakitani S., Shimamoto A., Kamei N., Sawa M., Adachi N., Ochi M. (2017). Magnetic Targeted Delivery of Induced Pluripotent Stem Cells Promotes Articular Cartilage Repair. Stem Cells Int..

[168] Li Z., Wang Y., Xiao K., Xiang S., Li Z., Weng X. (2018). Emerging Role of Exosomes in the Joint Diseases. Cell. Physiol. Biochem..

[169] Phinney D.G., Pittenger M.F. (2017). Concise Review: MSC-Derived Exosomes for Cell-Free Therapy. Stem Cells.

[170] Zhang S., Chuah S.J., Lai R.C., Hui J.H.P., Lim S.K., Toh W.S. (2018). MSC Exosomes Mediate Cartilage Repair by Enhancing Proliferation, Attenuating Apoptosis and Modulating Immune Reactivity. Biomaterials.

[171] Liu X., Li Q., Niu X., Hu B., Chen S., Song W., Ding J., Zhang C., Wang Y. (2017). Exosomes Secreted from Human-Induced Pluripotent Stem Cell-Derived Mesenchymal Stem Cells Prevent Osteonecrosis of the Femoral Head by Promoting Angiogenesis. Int. J. Boil. Sci..

[172] Lener T., Gimona M., Aigner L., Börger V., Buzas E., Camussi G., Chaput N., Chatterjee D., Court F.A., Del Portillo H.A. (2015). Applying Extracellular Vesicles Based Therapeutics in Clinical Trials—An ISEV Position Paper. J. Extracell. Vesicles.

[173] Baglío S.R., Pegtel D.M., Baldini N. (2012). Mesenchymal Stem Cell Secreted Vesicles Provide Novel Opportunities in (stem) Cell-Free Therapy. Front. Physiol..

[174] Zhang H.-C., Liu X.-B., Yang Y., Guo Z.-K., Huang S., Bi X.-Y., Wang H.-X., Xie L.-X., Wang Y.-Q., Cao X.-F. (2012). Microvesicles Derived from Human Umbilical Cord Mesenchymal Stem Cells Stimulated by Hypoxia Promote Angiogenesis Both In Vitro and In Vivo. Stem Cells Dev..

[175] Keshtkar S., Azarpira N., Ghahremani M.H. (2018). Mesenchymal Stem Cell-Derived Extracellular Vesicles: Novel Frontiers in Regenerative Medicine. Stem Cell Res. Ther..

[176] Théry C., Witwer K.W., Aikawa E., Alcaraz M.J., Anderson J.D., Andriantsitohaina R., Antoniou A., Arab T., Archer F., Atkin-Smith G.K. (2018). Minimal Information for Studies of Extracellular Vesicles 2018 (MISEV2018): A Position Statement of the International Society for Extracellular Vesicles and Update of the MISEV2014 Guidelines. J. Extracell. Vesicles.

[177] Lötvall J., Hill A.F., Hochberg F., Buzás E.I., Di Vizio L., Gardiner C., Gho Y.S., Kurochkin I.V., Mathivanan S., Quesenberry P. (2014). Minimal Experimental Requirements for Definition of Extracellular Vesicles and Their Functions: A Position Statement from the International Society for Extracellular Vesicles. J. Extracell. Vesicles.

